# Computational assessment of the radical scavenging activity of cleomiscosin†

**DOI:** 10.1039/d4ra03260h

**Published:** 2024-07-29

**Authors:** Trung Quang Nguyen, Adam Mechler, Quan V. Vo

**Affiliations:** a The University of Danang – University of Science and Education Da Nang 550000 Vietnam; b Quality Assurance and Testing Center 2 Da Nang 550000 Vietnam; c Department of Biochemistry and Chemistry, La Trobe University Victoria 3086 Australia; d Faculty of Chemical Technology – Environment, The University of Danang – University of Technology and Education Da Nang 550000 Vietnam vvquan@ute.udn.vn

## Abstract

Coumarinolignans such as cleomiscosin A (CMA), cleomiscosin B (CMB), and cleomiscosin C (CMC) are secondary metabolites that were isolated from diverse plant species. Cleomiscosins (CMs) have numerous interesting biological activities, including noteworthy cytotoxicity of cancer cell lines along with hepatoprotective and assumed antioxidant activities. In this present study, the antioxidant properties of three cleomiscosins were investigated with a focus on the structure–activity relationship using thermodynamic and kinetic calculations with the M06-2X/6-311++G(d,p) method. The results show that CMs, including CMA, CMB, and CMC, are weak antioxidants in apolar environments, with *k*_overall_ of 7.52 × 10^2^ to 6.28 × 10^4^ M^−1^ s^−1^ for the HOO˙ radical scavenging reaction in the gas phase and 3.47 × 10^2^ to 6.44 × 10^4^ M^−1^ s^−1^ in pentyl ethanoate. Remarkably, the difference in the fusion of phenylpropanoid structure with coumarin *via* two *ortho*-hydroxyl groups (CMA and CMB) does not cause any noticeable effect on their antioxidant activity, while the presence of a methoxy substitute on the aromatic ring of phenylpropanoid units (CMC) increases the reaction rate to about 61 to 84 times faster than that of CMA. In contrast, the studied CMs exhibit a good antioxidant capacity in polar environments, with a *k*_overall_ range from 4.03 × 10^7^ to 8.66 × 10^7^ M^−1^ s^−1^, 10^2^–10^3^ times faster than that of Trolox, equal to that of ascorbic acid and resveratrol. The angular fusion of the phenylpropanoid and coumarin structures, as well as the methoxy substitution on the aromatic ring of the phenylpropanoid unit of the studied CMs, do not have any considerable effect on their antioxidant activity under the studied conditions.

## Abstract 2

Cleomiscosins from traditional medicinal plants are promising radical scavengers in aqueous physiological environments.

## Graphical abstract

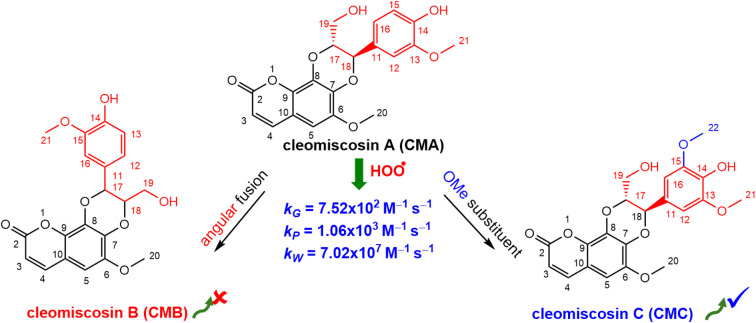

## 1. Introduction

Coumarinolignans (CLs) are secondary metabolites in a diverse range of plant species. The fusion of coumarin with phenyl propanoid structure yields many CL isomers. Numerous coumarinolignans were isolated from traditional medicinal plants, such as *Chloranthus japonicus* (Sieb.), *Terminalia tropophylla* (H.Perrier), *Artemisia minor* (Jacquem. ex Besser), *Sapium discolor*, *Zanthoxylum avicennae* (Lamk.), and *Brucea javanica* ((L.) Merr).^1–6^ Among them, cleomiscosin A (CMA), cleomiscosin B (CMB), and cleomiscosin C (CMC) are the chemical species found in most plant sources.^7–12^ Previous studies reported many pharmacologically important activities of cleomiscosin (CM) substances, with the most prominent being their anti-inflammatory,^13–15^ anti-cancer cytotoxic,^16–18^ anti-oxidant,^19–21^ and hepatoprotective activity.^22,23^

Some *in vitro* studies have documented the radical scavenging activity of select CM compounds. The substituent groups on the aromatic ring and the spatial position of the propanoid unit were found to influence the antioxidant activity of these compounds.^19–21^

**Fig. 1:**
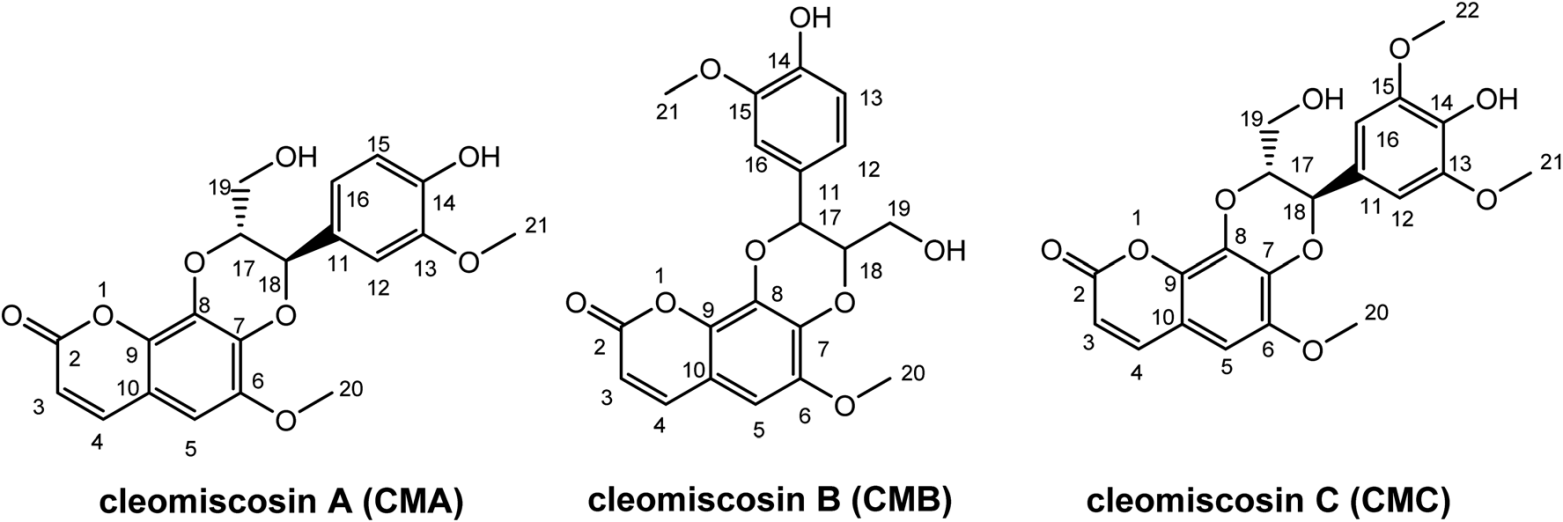
Structure of studied cleomiscosins.

Prior research widely used computational methods to study the link between structure and activity, and to guide the development of new medicines with enhanced activity. Consequently, they became powerful tools in the arsenal of medicinal chemistry.^24–29^ In this study, the antioxidant potentials of CMA, CMB, and CMC along with their structure–activity relationship were investigated, using the M06-2X/6-311++G(d,p) method. The structures and atom numbering of these compounds are presented in Fig. 1.

## 2. Computational details

All calculations in this study were performed using the Gaussian 16 suite of programs^30^ with the M06-2X/6-311++G(d,p) method,^31,32^ using the solvation model density (SMD) method (pentyl ethanoate for lipid and water for polar media).^33^ The good performance of the DFT/M06-2X functional in predicting the thermodynamic and kinetic parameters of radical scavenging reaction has been validated by previous studies.^34,35^ The antioxidant activity of studied compounds was evaluated using the quantum mechanics based test for overall free radical scavenging activity (QM-ORSA) protocol.^24,25,36–39^ The radical scavenging reaction rate constants were predicted using the conventional transition state theory (TST) and 1 M standard state at 298.15 K.^40–43^ The details of the calculating method can be found in Table S1, ESI.†

The following calculations were performed to determine the proton affinity (PA), ionization energy (IE), and bond dissociation enthalpy (BDE) that are the determinants of the likelihood of the reaction proceeding *via* either of the three primary mechanisms: sequential proton loss electron transfer (SPLET), sequential electron transfer proton transfer (SETPT), or formal hydrogen transfer (FHT).^26,44–47^

1 CM–OH → CM–O^−^ + H^+^; PA = *H*(CM–O^−^) + *H*(H^+^) − *H*(CM–OH)

2 CM–OH → CM–OH^+^˙ + e^−^; IE = *H*(CM–OH^+^˙) + *H*(e^−^) − *H*(CM–OH)

3 CM–OH → CM–O˙ + H˙; BDE = *H*(CM–O˙) + *H*(H˙) − *H*(CM–OH)

where *H*(H^+^), *H*(H˙), *H*(CM–OH), *H*(CM–O^−^), *H*(CM–O˙), and *H*(CM–OH^+^˙) are enthalpies of proton, hydrogen atom, neutral molecule, anion, radical and cation-radical, respectively. Eqn (4)–(6) were used to calculate the Gibbs free energies (Δ*G*°) for the first step of each possible pathway in the CM + HOO˙ reactions *e.g.* either proton transfer (PT), FHT or single electron transfer (SET) mechanisms.PT: CM–OH + HOO˙ → CM–O^−^ + HOOH˙^+^

4 Δ*G*° = *G*(CM–O^−^) + *G*(HOOH˙^+^) − *G*(CM–OH) − *G*(HOO˙)

FHT: CM–OH + HOO˙ → CM–O˙ + HOOH;

5 Δ*G*° = *G*(CM–O˙) + *G*(HOOH) − *G*(CM–OH) − *G*(HOO˙)

SET: CM–OH + HOO˙ → CM–OH˙^+^ + HOO^−^

6 Δ*G*° = *G*(CM–OH˙^+^) + *G*(HOO^−^) − *G*(CM–OH) − *G*(HOO˙)

where *G*(HOO^−^), *G*(HOO˙), *G*(CM–OH), *G*(CM–O^−^), *G*(CM–OH^+^˙), *G*(CM–O˙) and *G*(HOOH˙^+^) are Gibb energies of the HOO^−^, HOO˙, neutral molecule, anion, cation-radical, radical and HOOH˙^+^, respectively.

## 3. Results and discussion

### 3.1. The HOO radical scavenging of cleomiscosins in the gas phase

#### 3.1.1. Thermodynamic evaluation

The thermodynamic parameters of studied compounds in the gas phase were screened first by M06-2X/6-311++G(d,p) method, *via* BDE, IE and PA calculated values, corresponding to the first step of FHT, SETPT, and SPLET reaction pathways. The change of Gibbs free energy (Δ*G*°) of the first step of each pathway of the possible bond was calculated and shown in Table 1.

**Table 1:** Computed BDEs, IEs, PAs, in kcal mol^−1^ of the possible OH/CH bonds of cleomiscosins and Δ*G*° of the CM + HOO˙ reactions following the FHT, proton transfer (PT), and single electron transfer (SET) pathways in the gas phase

Positions	Mechanisms					
	FHT (kcal mol^−1^)		PT (kcal mol^−1^)		SET (kcal mol^−1^)	
	BDE	Δ*G*°	PA	Δ*G*°	IE	Δ*G*°
CMA–O14–H	88.5	3.3	340.6	187.8	182.4	159.6
CMA–C18–H	90.2	4.9	363.1	211.4		
CMA–O19–H	105.8	20.4	354.2	203.4		
CMB–O14–H	88.3	2.9	343.5	187.8	179.1	156.5
CMB–C17–H	89.7	4.1	364.2	211.4		
CMB–O19–H	105.4	19.7	353.5	203.4		
CMC–O14–H	84.2	−1.5	334.6	183.1	179.3	155.9
CMC–C18–H	86.3	0.1	358.2	205.8		
CMC–O19–H	106.1	20.6	364.0	211.7		

CMC has the lowest BDE and PA values, however the values are generally very similar, CMA and CMB are not significantly different. The O14–H bond has a lower BDE value than any other positions of the three studied compounds. CMC has the lowest BDE at 84.2 kcal mol^−1^ at O14–H position that is still higher than that of viniferifuran (82.7 kcal mol^−1^)^48^ or resveratrol (83.9 kcal mol^−1^).^48^ Additionally, the BDE of CMA–O14–H and CMB–O14–H are 88.5 and 88.3 kcal mol^−1^, respectively, higher than that of reference antioxidants. The results suggest that the antioxidant activity of these positions is weak in the absence of bond bond-weakening high dielectric medium. The PA values of studied compounds range from 334.6 kcal mol^−1^ to 364.2 kcal mol^−1^, and the IE values range from 179.1 kcal mol^−1^ to 182.4 kcal mol^−1^.

The results also show that the Gibbs free energy changes (Δ*G*°) of the first step of the CMC + HOO˙ reactions following the FHT mechanism are −1.5 and 0.1 kcal mol^−1^ corresponding to O14–H and C18–H position, while that of CMA and CMB range from 2.9 to 4.9 kcal mol^−1^. Hence this mechanism should be used for further investigation. Whereas, the radical scavenging reactions of CMs in the gas phase do not follow neither the SETPT nor SPLET pathway, due to the positive value of Δ*G*°, much higher than that of the FHT pathway. Thus, these pathways should be omitted in the kinetic calculation.

##### Kinetic study

**Fig. 2:**
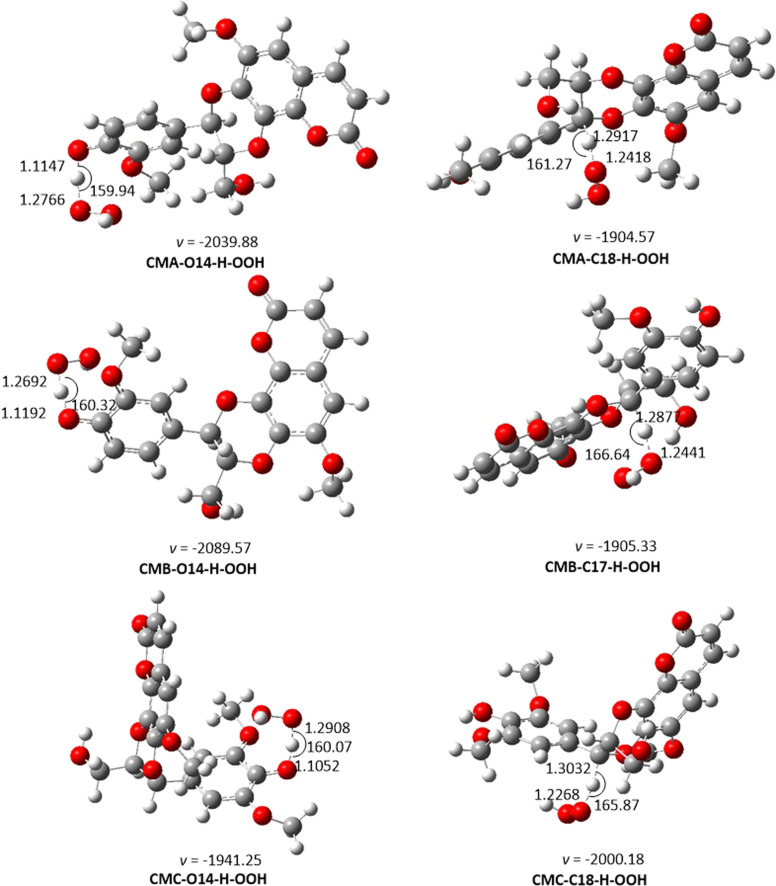
The TS-optimized structures of CM + HOO˙ reaction in the gas phase following the FHT pathway.

From the thermodynamic data, the preferred mechanism of CM + HOO˙ reactions in the gas phase is FHT. The kinetic parameters were predicted for O14–H and C–18H positions of the studied compounds. The results are shown in Table 2 and Fig. 2.

**Table 2:** Calculated activation Gibbs free energies (Δ*G*^‡^, kcal mol^−1^), tunneling corrections (*κ*), *k*_Eck_ (M^−1^ s^−1^) and branching ratios (*Γ*, %) of the FHT mechanism for the CM + HOO˙ reaction in the gas phase

Comp.	Mechanisms	Positions	Δ*G*^‡^ (kcal mol^−1^)	*κ*	*k*_Eck_ (M^−1^ s^−1^)	*Γ* (%)
CMA	FHT	O14–H	16.7	195.2	7.29 × 10^2^	96.9
		C18–H	18.6	153.0	2.30 × 10^1^	3.1
	*k*_overall_				7.52 × 10^2^	
CMB	FHT	O14–H	16.4	472.4	2.84 × 10^3^	98.1
		C17–H	18.3	209.3	5.42 × 10^1^	1.9
	*k*_overall_				2.90 × 10^3^	
CMC	FHT	O14–H	14.2	254.4	6.28 × 10^4^	100.0
		C18–H	20.1	912.1	1.04 × 10^1^	0.0
	*k*_overall_				6.28 × 10^4^	

From the calculated data, CMs are weak antioxidants in the gas phase, with *k*_overall_ ranging from 7.52 × 10^2^ to 6.28 × 10^4^ M^−1^ s^−1^. The low reaction rates are associated with the high BDE value in the thermodynamic section. Thus, the O14–H location is the primary factor determining the radical scavenging activity of CMs in the gas phase. The *k*_overall_ of CMC + HOO˙ reactions is 6.28 × 10^4^, higher than that of CMA and CMB. This result is in logical agreement with the lowest BDE of CMC in the thermodynamic calculation. It was remarkable that the difference in the fusion of phenylpropanoid unit with coumarin moiety *via* two *ortho*-hydroxyl groups (CMA and CMB) causes an imperceptible change in their antioxidant activity, while the presence of an additional methoxy group on the aromatic ring of phenylpropanoid unit (CMC) increase the reaction rate about 84 times faster than that of CMA.

### 3.2. The HOO radical scavenging of cleomiscosins in the physiological environments

#### 3.2.1. Thermodynamic evaluation

Similarly to the previous section, the thermodynamic parameters of cleomiscosin were computed in pentyl ethanoate and water (pH = 7.4) and the results are listed in Tables 3 and 4.

**Table 3:** The computed thermodynamic data (BDE, IE, PA, in kcal mol^−1^) of CM and Δ*G*° of the primary mechanisms of the CM + HOO˙ reactions in the lipid medium

Positions	Mechanisms					
	FHT (kcal mol^−1^)		PT (kcal mol^−1^)		SET (kcal mol^−1^)	
	BDE	Δ*G*°	PA	Δ*G*°	IE	Δ*G*°
CMA–O14–H	86.0	0.5	308.3	105.9	146.2	75.2
CMA–C18–H	90.9	5.7	337.3	135.4		
CMA–O19–H	104.9	19.5	324.7	123.9		
CMB–O14–H	85.9	0.9	309.1	107.0	144.6	74.3
CMB–C17–H	90.9	6.0	338.1	136.5		
CMB–O19–H	104.8	19.7	324.0	122.6		
CMC–O14–H	84.0	−4.9	306.4	102.6	147.6	73.5
CMC–C18–H	87.4	−0.7	333.0	128.7		
CMC–O19–H	105.8	18.2	328.5	125.6		

**Table 4:** The predicted thermodynamic data (BDE, IE, PA, in kcal mol^−1^) of CM and Δ*G*° of the primary mechanisms of the CM + HOO˙ reactions in water

Positions	Mechanisms					
	FHT (kcal mol^−1^)		PT (kcal mol^−1^)		SET (kcal mol^−1^)	
	BDE	Δ*G*°	PA	Δ*G*°	IE	Δ*G*°
CMA–O14–H	85.6	−3.2	43.7	36.9	110.9	31.4
CMA–C18–H	93.2	4.2	83.1	76.2		
CMA–O19–H	107.0	18.0	56.0	49.4		
CMB–O14–H	85.6	−3.5	43.8	36.7	110.7	30.9
CMB–C17–H	93.6	4.4	83.9	76.5		
CMB–O19–H	107.1	17.8	55.9	49.2		
CMC–O14–H	86.9	−1.4	43.7	37.4	117.6	38.2
CMC–C18–H	91.0	2.4	79.3	72.1		
CMC–O19–H	107.1	19.4	58.9	52.3		

Pentyl ethanoate medium (Table 3) yields a similar trend to the gas phase, with BDE and PA values of CMA nearly equal to that of CMB, but higher than CMC. The IE value of CMB is lower than that of CMA and CMC. All thermodynamic parameters are slightly lower than in the gas phase. Particularly, BDE of O14–H of CMC is the lowest value at 84.0 kcal mol^−1^, and BDE of C18–H position is 87.4 kcal mol^−1^, while that of CMA and CMB ranges from 85.9 to 86.0 kcal mol^−1^ and 90.9 kcal mol^−1^, respectively. The PA values of studied compounds range from 306.4 to 338.1 kcal mol^−1^, while their IE values range from 144.6 to 147.6 kcal mol^−1^.

From the calculated Δ*G*° of the first step of each pathway, the HOO˙ radical scavenging reactions of CMC at O14–H and C18–H positions are energetically favored following the FHT pathway due to the Δ*G*° < 0. Conversely the SPLET and SETPT mechanisms are not preferred with the Δ*G*° much higher than that of FHT. Thus, only the kinetics of the FHT pathway of O14–H and C18–H should be calculated in pentyl ethanoate in the next section.

In contrast to the trend in apolar environments, the BDE values of O14–H of CMA and CMB in water (pH = 7.4) are 85.6 kcal mol^−1^, lower than that of CMC (86.9 kcal mol^−1^). Whereas, the C18–H position of CMC has the BDE value at 91.0 kcal mol^−1^, lower than that of CMA–C18–H and CMB–C17–H. The Gibbs free energy changes of the first step of CMs–O14–H + HOO˙ reactions following the FHT mechanism are negative, suggesting that these reactions are spontaneous, while the SETPT pathway is not favorable due to a much more positive Δ*G*° value.

**Fig. 3:**
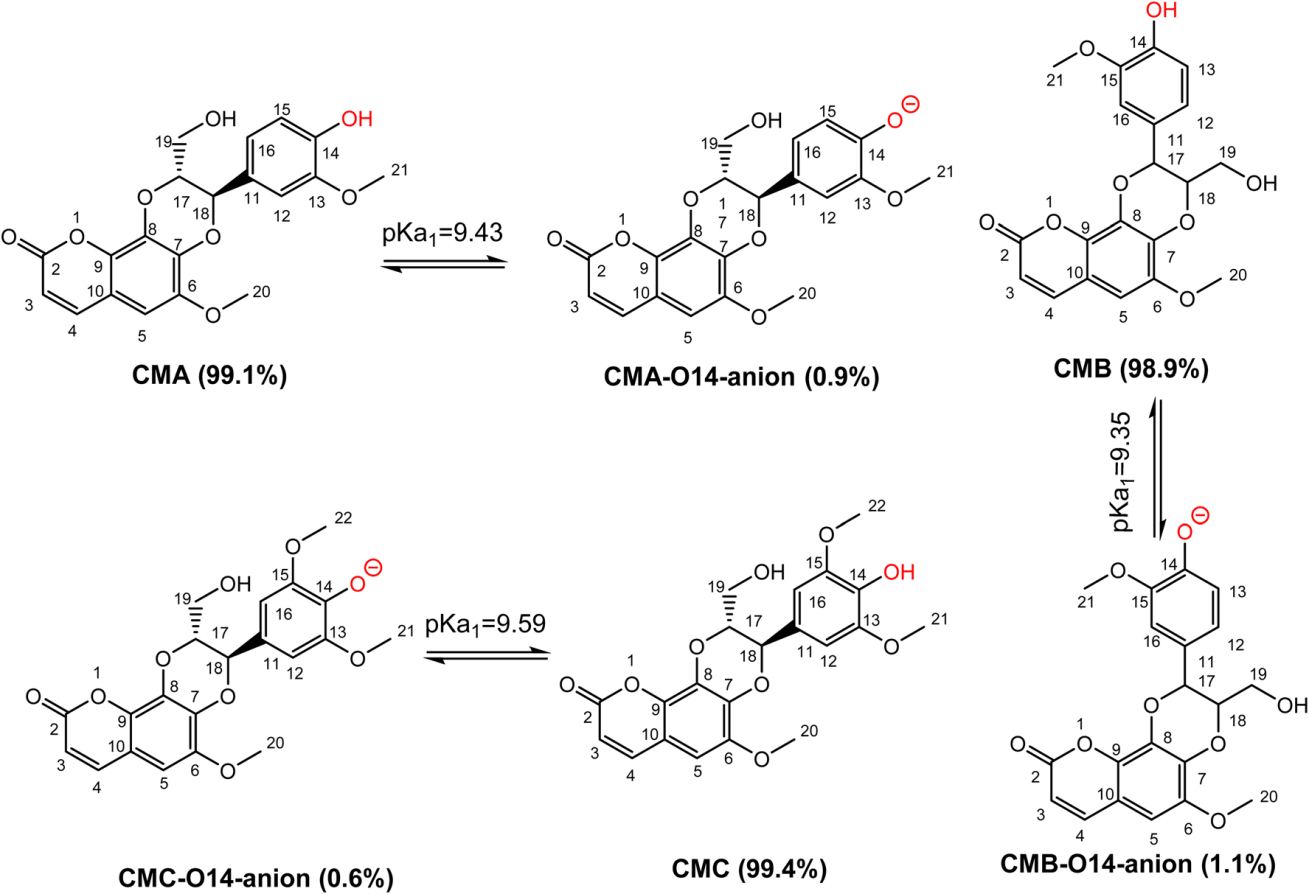
The deprotonation of cleomiscosins in water at pH = 7.4.

Previous studies showed that SPLET is the main mechanism of antioxidant activity of phenolic compounds in water at pH 7.4.^26,34^ The spontaneous dissociation of acidic protons in those substances in water eliminates the activation energy barrier of the first stage of reaction (PL), bringing the reaction directly to the second stage (SET).^25,26^ Thus, the contribution of each deprotonation state should be investigated. From calculated data, the PA values of the O14–H position of CMs are nearly equal, ranging from 43.7 kcal mol^−1^ to 43.8 kcal mol^−1^. In addition to that, the first deprotonation should take place at O14–H, followed by the O19–H position of CMs. Due to the lack of experiment p*K*_a_ values of studied compounds, these values were calculated following the ref. 49 and presented in Fig. 3.

The p*K*_a1_ of CMs are not significantly different, with values at pH 7.4 of CMA, CMB, and CMC are 9.43, 9.35, and 9.59, respectively. The populations of states of CMA are 99.1% neutral (HA) and 0.9% anion (A^−^), for CMB 98.9% are neutral (HA) and 1.1% are anion (A^−^), while for CMC 99.4% are neutral (HA) and 0.6% are anion (A^−^). These states should be used for kinetic investigation.

#### 3.2.2. Kinetic study

**Fig. 4:**
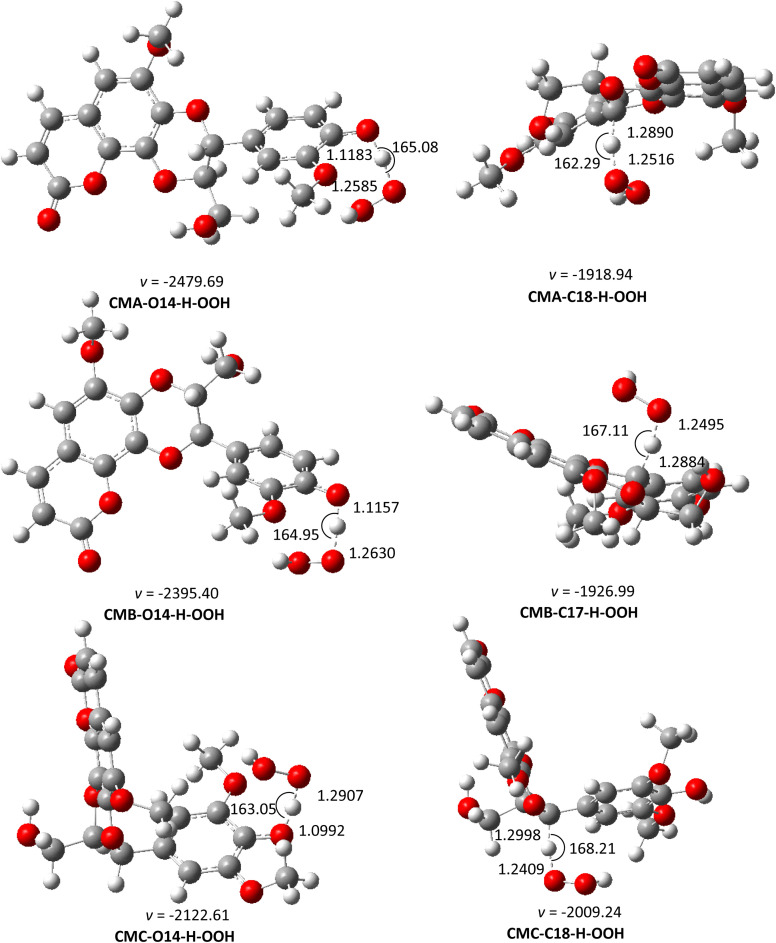
The optimized structures of TSs of the CM + HOO˙ reaction in pentyl ethanoate following the FHT pathway.

**Fig. 5:**
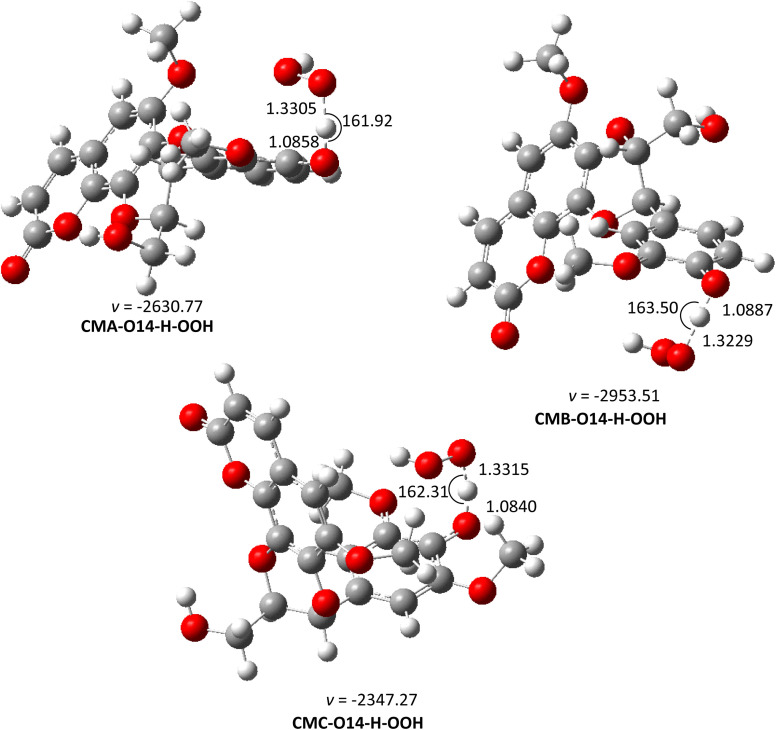
The optimized structures of TSs of the CM + HOO˙ reaction in water (pH = 7.4) following the FHT pathway.

From the thermodynamic parameter in solvents, the HOO˙ radical scavenging reaction of CMs in pentyl ethanoate was calculated following FHT pathway, while both FHT and SET mechanisms were computed in water (pH = 7.4). The results of the kinetic study in pentyl ethanoate are shown in Fig. 4 and Table 5, and that of HOO˙ radical scavenging reaction in water are presented in Fig. 5 and Table 6. The overall reaction rate constant was computed following the QM-ORSA protocol,^24,38^ according to eqn (7) and (8):

**Table 5:** Calculated activation Gibbs free energies (Δ*G*^‡^, kcal mol^−1^), tunneling corrections (*κ*), *k*_app_ (M^−1^ s^−1^) and branching ratios (*Γ*, %) for the HOO˙ scavenging of the CM in pentyl ethanoate following the FHT mechanism

Comp.	Mechanisms	Positions	Δ*G*^‡^ (kcal mol^−1^)	*κ*	*k*_app_ (M^−1^ s^−1^)	*Γ* (%)
CMA	FHT	O14–H	18.1	3224.1	1.06 × 10^3^	100.0
		C18–H	22.4	227.5	5.91 × 10^−2^	0.0
	*k*_overall_				1.06 × 10^3^	
CMB	FHT	O14–H	18.6	2311.7	3.47 × 10^2^	100.0
		C17–H	22.6	258.6	4.14 × 10^−2^	0.0
	*k*_overall_				3.47 × 10^2^	
CMC	FHT	O14–H	14.5	428.7	6.43 × 10^4^	100.0
		C18–H	19.3	743.7	3.20 × 10^1^	0.0
	*k*_overall_				6.44 × 10^4^	

**Table 6:** The calculated Δ*G*^‡^ (in kcal mol^−1^), tunneling corrections (*κ*), branching ratios (*Γ*, %) and rate constants (*k*_app_, *k*_f_, *k*_overall_, M^−1^ s^−1^) for HOO˙ scavenging of the cleomiscosins in the aqueous solution^a^

Comp.	Mechanisms	States	Δ*G*^‡^ (kcal mol^−1^)	*κ*	*k*_app_ (M^−1^ s^−1^)	*f*	*k*_f_ (M^−1^ s^−1^)	*Γ* (%)
CMA	FHT (O14)	HA	16.6	1664.7	6.99 × 10^3^	0.991	6.93 × 10^3^	0.0
	SET	HA	62.1	7.7*	1.90 × 10^−33^	0.991	1.88 × 10^−33^	0.0
	SET (O14)	A^−^	2.3 (4.5)	5.0* (7.4)	7.80 × 10^9^ (2.4 × 10^9^)	0.009	7.02 × 10^7^ (2.16 × 10^7^)	100.0
	*k*_overall_						7.02 × 10^7^	
CMB	FHT (O14)	HA	17.0	12 304.8	2.58 × 10^4^	0.989	2.56 × 10^4^	0.0
	SET	HA	59.7	8.1*	1.00 × 10^−31^	0.989	9.89 × 10^−32^	0.0
	SET (O14)	A^−^	2.4 (4.9)	5.4* (6.5)	7.80 × 10^9^ (1.30 × 10^9^)	0.011	8.66 × 10^7^ (1.43 × 10^7^)	100.0
	*k*_overall_						8.66 × 10^7^	
CMC	FHT (O14)	HA	18.7	865.5	1.13 × 10^2^	0.994	1.12 × 10^2^	0.0
	SET	HA	58.4	5.5*	9.30 × 10^−31^	0.994	9.24 × 10^−31^	0.0
	SET (O14)	A^−^	3.3 (5.0)	4.9* (8.1)	6.30 × 10^9^ (1.10 × 10^9^)	0.006	4.03 × 10^7^ (6.60 × 10^6^)	100.0
	*k*_overall_						4.03 × 10^7^	

a *k*_f_ = *f* × *k*_app_; *Γ* = *k* × 100/*k*_overall_; * the nuclear reorganization energy (*λ*, in kcal mol^−1^); in bracket are values for the SET reaction with the CM–O^−^·H_2_O/CM–O˙·H_2_O model.

In the pentyl ethanoate:

7 *k*_overall_ = ∑*k*_app_(FHT-neutral)

In water:

8 *k*_overall_ = ∑*k*_app_(FHT-neutral) + ∑*k*_app_(SET-neutral) + ∑*k*_app_(SET-anion)

From the data of Table 5, the *k*_overall_ of CMC in pentyl ethanoate is about 61 to 185 times faster than that of CMA and CMB. The *k*_overall_ of CMA and CMB are 1.06 × 10^3^ and 3.47 × 10^2^ M^−1^ s^−1^, respectively. These results suggest that CMs are not good antioxidants in non-polar solvents. Besides, the antioxidant activity of CMA, CMB, and CMC in that solvent is dominated by the O14–H position, while the C18–H bonds do not make any contribution (*Γ* ∼ 0.0%). Similarly to the trend in the gas phase, the difference in the fusion of phenylpropanoid unit with coumarin (CMA and CMB) does not cause any significant change in their antioxidant activities, whereas the presence of the methoxy group on the aromatic ring of phenylpropanoid unit increases the reaction rate by roughly 61 times.

As per calculated data in water at pH = 7.4, the fusion of phenylpropanoid unit and coumarin moiety, as well as methoxy substitution on the aromatic ring of phenylpropanoid unit do not have any effects on the protonation of studied coumarinolignans and their radical scavenging reaction rate. Particularly, the *k*_overall_ of CMs ranges from 4.03 × 10^7^ to 8.66 × 10^7^ M^−1^ s^−1^. The molar fraction value of the anion state (A^−^) of CMs is only 0.6–1.1%, but it contributes 100.0% of the reaction rate of CMs with HOO˙ radical, whereas the neutral states (HA) make almost no contribution at all to the overall reaction rate. It indicates that the antiradical activity of phenolics is significantly influenced by the phenoxide anion, despite the fact that this form is present in relatively small quantities in the polar environment. This outcome is consistent with prior studies.^26,50^

The CM–O^−^·H_2_O/CM–O˙·H_2_O model was also employed to investigate the impact of the explicit presence of a solvent, specifically a water molecule, on the HOO˙ radical scavenging of the primary mechanism (the SET reaction of anion states) (Table 6). In comparison to the reaction that did not contain the H_2_O molecule (*k*_overall_ = 4.03 × 10^7^ to 8.66 × 10^7^ M^−1^ s^−1^), the *k*_overall_ values can be reduced by 3.25–6.11 times in the presence of the H_2_O molecule in the phenoxide anions/radicals (*k*_overall_ = 6.06 × 10^6^ to 2.16 × 10^7^).

The reduction in the rate constant of the anion state following the SET mechanism may result from fluctuations in ionization energy that were observed in previous studies.^51,52^ This could be attributed to the interaction of a water molecule forming a hydrogen bond with the anion, leading to an increase in the molecule's ionization energy. As a result, the electron transfer reaction from the phenoxide anions to the HOO˙ free radical is hindered. These reaction rates are approximately 10^2^–10^3^ times faster than that of Trolox (*k* = 8.96 × 10^4^ M^−1^ s^−1^ (M05-2X/6-31+G(d,p)),^27^*k* = 1.30 × 10^5^ M^−1^ s^−1^ (M06-2X/6-311++G(d,p))^53^) and fairly similar to ascorbic acid (*k* = 9.97 × 10^7^ M^−1^ s^−1^, M05-2x/6-311++G(d,p))^24^ and resveratrol (*k* = 5.62 × 10^7^ M^−1^ s^−1^, M05-2X/6-311++G(d,p)).^50,54^ Thus the CMs are promising antioxidants in polar environments.

## 4. Conclusion

The HOO˙ radical scavenging activity of three coumarinoligans CMA, CMB, and CMC were calculated using M06-2X/6-311++G(d,p) method, and their structure–activity relationship was evaluated as well. The results show that these coumarinoligans are only weak antioxidants in non-polar environments, but they perform well in polar environments, with *k*_overall_ range from 4.03 × 10^7^ to 8.66 × 10^7^ M^−1^ s^−1^, mainly exerted by the anion states, *via* SET mechanism. The differences in the structure of these coumarinoligans do not affect antioxidant activity in polar environments, but the methoxy substitution on the aromatic ring of lignan moiety (CMC) increases the radical reaction rate in apolar environments by 61 to 84 times.

## Data availability

The data supporting this article have been included as part of the ESI.†

## Conflicts of interest

There are no conflicts to declare.

## Supplementary Material

RA-014-D4RA03260H-s001
