# Supplementary material for: Computational assessment of the radical scavenging activity of cleomiscosin
Source: RSC Adv. 2024 Jul 29;14(33):23629–37. doi: 10.1039/d4ra03260h (PMC11284531; doi:10.1039/d4ra03260h)
Supplement: RA-014-D4RA03260H-s001 [file RA-014-D4RA03260H-s001.pdf]

Supplementary Information (SI) for RSC Advances.
This journal is © The Royal Society of Chemistry 2024

Supporting Information (SI)

Computational assessment of the radical scavenging activity of natural cleomiscosins

Nguyen Quang Trung,[1,2] Adam Mechler[3] and Quan V. Vo[4*]

[1]*The University of Danang - University of Science and Education, Da Nang 550000, Vietnam.*

[2]*Quality assurance and Testing center 2, Da Nang 550000, Vietnam.*

[3]*Department of Biochemistry and Chemistry, La Trobe University, Victoria 3086, Australia.*

[4]*The University of Danang - University of Technology and Education, Danang 550000, Vietnam.*

*Corresponding authors: vvquan@ute.udn.vn;

Table of Contents

Table S1. The method to calculate rate constant following the conventional transition state theory 2

Table S2: The Cartesian coordinates and energies of CMs, ANION, TSs in the studied media (G: gas phase; P: pentyl ethanoate; H: water) 3

References 4

Table S1. The method to calculate rate constant following the conventional transition state theory

The rate constant (k) was calculated by using the conventional transition state theory (TST) (at 298.15 K, 1M standard state) according to the equation (1):[1-5]

$$k = \sigma\kappa\frac{k_B T}{h}e^{-(\Delta G^{\neq})/RT} \quad (1)$$

Where: σ is the reaction symmetry number ,[6,7]
κ contains the tunneling corrections calculated using the Eckart barrier,[8]
k_B is the Boltzmann constant,
h is the Planck constant,
$\Delta G^{\neq}$ is the Gibbs free energy of activation.

The Marcus Theory was used to estimate the reaction barriers of SET reactions.[9-12] The free energy of reaction $\Delta G^{\neq}$ for the SET pathway was computed following the equations (2,3).

$$\Delta G^{\neq}_{SET} = \frac{\lambda}{4}\left(1 + \frac{\Delta G^{0}_{SET}}{\lambda}\right)^2 \quad (2)$$

$$\lambda \approx \Delta E_{SET} - \Delta G^{0}_{SET} \quad (3)$$

where ΔG_{SET} is the Gibbs energy of reaction, ΔE_{SET} is the non-adiabatic energy difference between reactants and vertical products for SET ($\Delta E_{SET} = E_{vertical}$ (products) - $E_{adiabatic}$ (reactants))[13,14]

For rate constants that were close to the diffusion limit a correction was applied to yield realistic results[15]. The apparent rate constants (k_{app}) were calculated following the Collins–Kimball theory in the solvents at 298.15K;[16] the steady-state Smoluchowski rate constant (k_D) for an irreversible bimolecular diffusion–controlled reaction was calculated following the literature as corroding to equations (4,5).[15,17]

$$k_{app} = \frac{k_{TST}k_D}{k_{TST} + k_D} \quad (4)$$

$$k_D = 4\pi R_{AB} D_{AB} N_A \quad (5)$$

where R_{AB} is the reaction distance, N_A is the Avogadro constant, and $D_{AB} = D_A + D_B$ (D_{AB} is the mutual diffusion coefficient of the reactants A and B),[16,18] where D_A or D_B is estimated using the Stokes–Einstein formulation (6).[19,20]

$$D_{A\ or\ B} = \frac{k_B T}{6\pi\eta a_{A\ or\ B}} \quad (6)$$

η is the viscosity of the solvents (i.e. $\eta(H_2O) = 8.91\times10^{-4}$ Pa s, η(pentyl ethanoate) = 8.62×10^{-4} Pa s) and a is the radius of the solute.

The kinetic study requires different considerations. Water and pentyl ethanoate are the *de facto* standard solvents in the literature to mimic the polar and nonpolar environments in the human body.[15,21-23] Thus, these solvents were used to model the physiological environments. The solvent cage effects were included following the corrections proposed by Okuno,[24] adjusted with the free volume theory according to the Benson correction[15,25-27] to reduce over-penalizing entropy losses in solution. For the species that have multiple conformers, all of these were investigated and the conformer with the lowest electronic energy was included in the analysis.[22,23] The hindered internal rotation treatment was also applied to the single bonds to ensure that the obtained conformer has the lowest electronic energy.[23,28] All transition states were characterized by the existence of only one single imaginary frequency. Intrinsic coordinate calculations (IRCs) were performed to ensure that each transition state is connected correctly with the pre-complex and post-complex. The kinetic calculations were performed by the *Eyringpy* code[29,30]

Table S2: The Cartesian coordinates **and energies** of CMs, ANIONs, TSs in the studied media (G: gas phase; P: pentyl ethanoate; H: water)

**Name**				**CMA-G**
Cartesian Coordinates				Energy
O	0.08395500	1.22354200	-0.04499100	Zero-point correction= 0.359109 (Hartree/Particle)
O	-1.11653100	-1.31339600	-0.34213000	Thermal correction to Energy= 0.383556
O	1.01400800	-2.85686400	0.68803600	Thermal correction to Enthalpy= 0.384500
O	-3.76529400	-1.54519900	-0.20653200	Thermal correction to Gibbs Free Energy= 0.304077
O	-1.48017200	3.44836100	0.20367800	Sum of electronic and zero-point Energies= -1374.218534
O	5.62901500	-0.96540500	0.75873900	Sum of electronic and thermal Energies= -1374.194087
O	6.27932900	0.85250200	-1.04229900	Sum of electronic and thermal Enthalpies= -1374.193142
O	-5.49944000	-2.91612300	-0.27363300	Sum of electronic and thermal Free Energies= -1374.273565
C	0.82894300	0.01794800	0.14942600	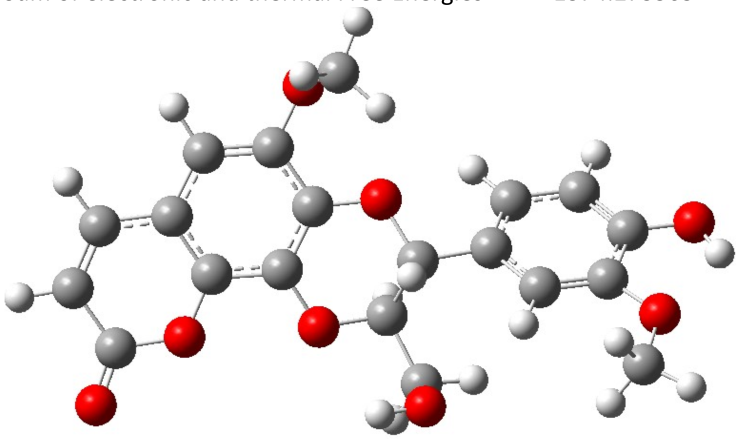
C	0.23147600	-1.06907300	-0.74910000	
C	2.27580700	0.29251100	-0.15065000	
C	-1.85213600	-0.18811400	-0.14641500	
C	-1.26258400	1.06560600	-0.01176000	
C	0.93450400	-2.40929900	-0.65124500	
C	-3.24479200	-0.29809700	-0.08999100	
C	-2.06265600	2.21708300	0.14165200	
C	-4.04112000	0.83387900	0.06719200	
C	3.24874300	-0.46986700	0.50782700	
C	2.65336600	1.23399800	-1.09663800	
C	-3.43378400	2.09429000	0.17744800	
C	4.58585800	-0.28610700	0.20205100	
C	4.00295300	1.42582500	-1.39328400	
C	-5.46783200	0.62472500	0.09728300	
C	4.96892400	0.67098500	-0.75410000	
C	-5.98352700	-0.61065400	-0.01637100	
C	-5.12448900	-1.78455200	-0.17345800	
C	-0.78747300	3.71694800	1.42111300	
C	5.31975200	-1.96983100	1.70808400	
H	0.70985500	-0.30259200	1.19042800	
H	0.23878200	-0.70969600	-1.78658900	
H	0.39689900	-3.12789200	-1.27786100	
H	1.95549100	-2.31707300	-1.02132000	
H	2.93761600	-1.21224900	1.23389000	
H	1.89796700	1.83291700	-1.58979800	
H	-4.03537200	2.98868000	0.28670300	
H	4.32123800	2.16117000	-2.12168600	
H	-6.11589300	1.48737800	0.21270400	
H	0.11614400	-3.02236500	0.99410400	
H	-7.04549800	-0.81162600	0.00130400	
H	-0.41694700	4.73689500	1.34175700	
H	0.05109200	3.03117300	1.55302700	
H	-1.47458800	3.64010800	2.26912700	
H	6.79330500	0.24073400	-0.50141800	
H	4.82274200	-1.53753900	2.58100500	
H	4.67976000	-2.73886900	1.26615600	
H	6.26884300	-2.40770600	2.00715200	
**Name**				**CMB-G**
Cartesian Coordinates				Energy
O	0.07256800	0.31177600	-0.28937800	Zero-point correction= 0.359150 (Hartree/Particle)
O	1.04501500	-2.28917600	0.19781000	Thermal correction to Energy= 0.383540

O	1.77236600	2.35619800	-0.41070600	Thermal correction to Enthalpy= 0.384484
O	-0.95562500	-3.72020300	-1.15596400	Thermal correction to Gibbs Free Energy= 0.304214
O	3.72899200	-2.73753200	0.32788900	Sum of electronic and zero-point Energies= -1374.217304
O	-4.42939700	1.92147100	1.20118600	Sum of electronic and thermal Energies= -1374.192914
O	-6.18160900	0.52212300	-0.18833500	Sum of electronic and thermal Enthalpies= -1374.191970
O	2.00941200	4.54322000	-0.63820300	Sum of electronic and thermal Free Energies= -1374.272239
C	-0.71922300	-0.83868100	-0.59388400	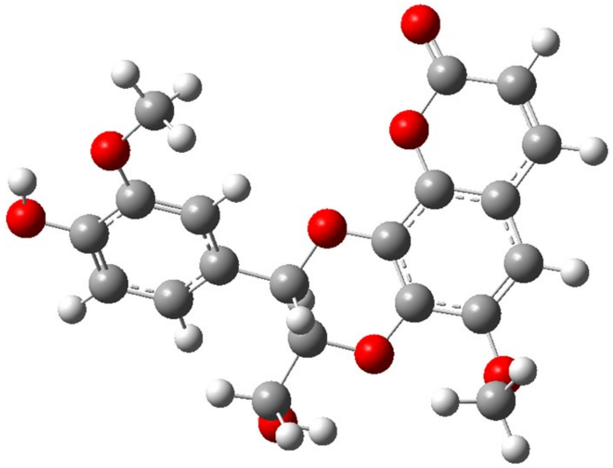
C	-0.33777900	-1.95599500	0.38272100	
C	-2.17035200	-0.45145800	-0.50290300	
C	1.39515900	0.05546300	-0.14721900	
C	1.88337300	-1.22773100	0.07738200	
C	-1.08874400	-3.25695400	0.17068200	
C	2.29930100	1.12039000	-0.22723500	
C	3.26974800	-1.46120100	0.17206200	
C	3.67117300	0.89926900	-0.12791500	
C	-2.56649700	0.59156500	0.33964900	
C	-3.11991200	-1.15344500	-1.23481100	
C	4.14817300	-0.40645800	0.06616800	
C	-3.90973600	0.91713800	0.43778500	
C	-4.47069400	-0.83262000	-1.12314600	
C	4.52541200	2.05613000	-0.24243300	
C	-4.87076100	0.19816600	-0.29071200	
C	4.00274400	3.28055100	-0.42138100	
C	2.55644400	3.48807600	-0.50225700	
C	3.57185500	-3.25331100	1.64671300	
C	-3.51755500	2.75667800	1.89175200	
H	-0.47938300	-1.17462700	-1.60837200	
H	-0.48490500	-1.59084800	1.40766500	
H	-0.71187800	-3.99384100	0.88862100	
H	-2.15170100	-3.10434400	0.35496300	
H	-1.81408000	1.15418300	0.87560300	
H	-2.80547200	-1.95922500	-1.89006000	
H	5.21197800	-0.60144100	0.13085500	
H	-5.22650300	-1.36484400	-1.68703800	
H	5.59962600	1.91541900	-0.18121900	
H	-0.02147700	-3.89064500	-1.31538200	
H	4.60570200	4.17329800	-0.51063900	
H	3.97815000	-4.26271400	1.63304500	
H	2.51734200	-3.28498800	1.92935800	
H	4.13045800	-2.64095600	2.36089100	
H	-6.25703300	1.27566600	0.40968900	
H	-4.11801600	3.50981000	2.39601800	
H	-2.95111600	2.18425500	2.63173400	
H	-2.82824400	3.23939500	1.19377600	
**Name**				**CMC-G**
Cartesian Coordinates				Energy
O	-0.13292900	-2.21086700	0.43785400	Zero-point correction= 0.391894 (Hartree/Particle)
O	1.39941600	-0.14676600	1.60267800	Thermal correction to Energy= 0.419069
O	2.37179900	-1.57807400	3.79718400	Thermal correction to Enthalpy= 0.420013
O	3.24585500	1.10096300	0.16986800	Thermal correction to Gibbs Free Energy= 0.333301
O	0.22808200	-2.82507300	-2.24046700	Sum of electronic and zero-point Energies= -1488.687765
O	-2.00975000	3.17412600	0.27776800	Sum of electronic and thermal Energies= -1488.660590
O	-5.03865300	-0.39655800	-0.25797200	Sum of electronic and thermal Enthalpies= -1488.659646
O	-4.37147600	2.29469700	-0.48000800	Sum of electronic and thermal Free Energies= -1488.746358

O	4.80175200	2.67181000	0.14118700
C	-0.65710100	-1.46266000	1.55222600
C	0.51353600	-0.95762400	2.38828900
C	-1.62906000	-0.41184000	1.06123200
C	1.56202200	-0.52925500	0.30710900
C	0.78996400	-1.52467300	-0.28586200
C	1.32777100	-2.08785600	3.00389300
C	2.53311100	0.12602400	-0.45311400
C	0.97164600	-1.85801700	-1.64258900
C	-2.88042300	-0.84095400	0.63355800
C	-1.28796200	0.94031700	0.95624300
C	2.74659900	-0.22487300	-1.78409000
C	1.95505300	-1.22137900	-2.37139000
C	-2.20950700	1.83449600	0.43013600
C	-3.80411200	0.05507300	0.10403300
C	-3.47377100	1.40572600	0.00593000
C	3.78203900	0.49462500	-2.48587000
C	4.48737200	1.45811300	-1.87018300
C	4.23318000	1.81795200	-0.47477100
C	-1.19244400	-2.71226500	-2.12851500
C	-0.75132500	3.69989100	0.66705200
C	-5.32874600	-0.29313800	-1.64969600
H	-1.19963200	-2.20768600	2.13688800
H	0.15448800	-0.29677700	3.17989200
H	0.68874200	-2.68231900	3.66005900
H	1.70706900	-2.74150700	2.20798800
H	-3.16951100	-1.88403100	0.70368000
H	-0.31785800	1.28925700	1.28120800
H	2.09313800	-1.49611100	-3.40990400
H	3.97870300	0.24018800	-3.52221400
H	2.88953800	-0.97422000	3.25405500
H	5.27057300	2.02055600	-2.35889000
H	-1.60355600	-3.11240800	-3.05446600
H	-1.48978300	-1.66602800	-2.01219600
H	-1.55793300	-3.28504600	-1.27694700
H	-3.96569700	3.17041500	-0.44780800
H	-0.58178400	3.54838800	1.73623500
H	-0.78810000	4.76425600	0.44961300
H	0.05735000	3.23435500	0.09700100
H	-6.31894200	-0.72290600	-1.78637300
H	-4.59765700	-0.86631500	-2.22904600
H	-5.32898100	0.74779500	-1.97524800

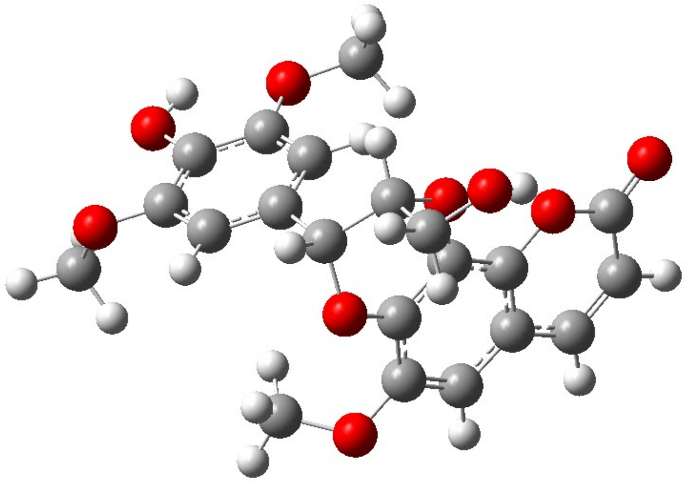

**Name**	**CMA-P**
Cartesian Coordinates	Energy

O	0.09574500	1.19540000	-0.05276900	Zero-point correction=	0.358295 (Hartree/Particle)
O	-1.13200600	-1.33200300	-0.33025400	Thermal correction to Energy=	0.382904
O	0.98412600	-2.89452700	0.72589800	Thermal correction to Enthalpy=	0.383849
O	-3.78838700	-1.52682800	-0.20359200	Thermal correction to Gibbs Free Energy=	0.302844
O	-1.42899400	3.42982900	0.19672800	Sum of electronic and zero-point Energies=	-1374.247999
O	5.65662800	-0.84188400	0.83346800	Sum of electronic and thermal Energies=	-1374.223390
O	6.26929700	0.78922600	-1.14735200	Sum of electronic and thermal Enthalpies=	-1374.222446
O	-5.53000600	-2.87810500	-0.27233300	Sum of electronic and thermal Free Energies=	-1374.303450
C	0.83538000	-0.01803600	0.14317900		
C	0.22453000	-1.11056900	-0.73800800		

C	2.27817700	0.24806700	-0.18529300	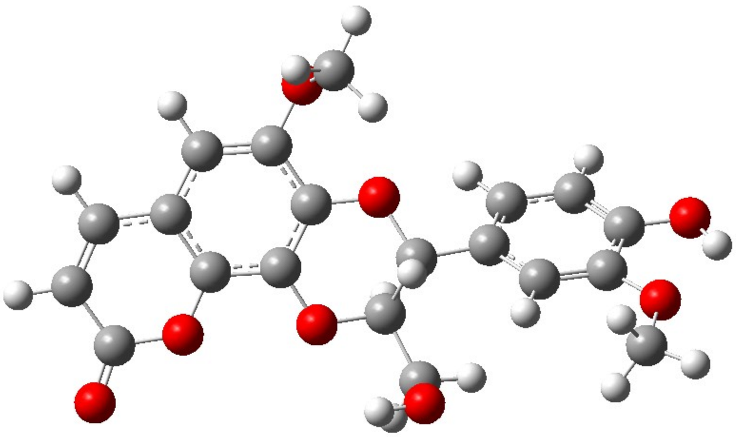
C	-1.85271700	-0.19796300	-0.14550800	
C	-1.24948500	1.05030600	-0.01717800	
C	0.91308100	-2.45574100	-0.61867100	
C	-3.24678300	-0.28584300	-0.08936600	
C	-2.03776100	2.21130600	0.13209100	
C	-4.03168600	0.85437700	0.06522300	
C	3.26728500	-0.42561100	0.54120300	
C	2.63536100	1.09315600	-1.22693700	
C	-3.41022700	2.10884600	0.17113500	
C	4.60176900	-0.24847300	0.21409900	
C	3.98027500	1.27946000	-1.54859700	
C	-5.45951900	0.66401500	0.09861100	
C	4.96178600	0.61271900	-0.83767200	
C	-5.99119900	-0.56681000	-0.01210400	
C	-5.14539700	-1.74326700	-0.16980400	
C	-0.82463000	3.70732800	1.46230100	
C	5.37159100	-1.74307100	1.89534400	
H	0.73327500	-0.32069800	1.19056600	
H	0.23633500	-0.77433700	-1.78251200	
H	0.37403500	-3.17649400	-1.24197600	
H	1.93548300	-2.37850600	-0.98918700	
H	2.97737400	-1.09263200	1.34432300	
H	1.87029000	1.62236600	-1.78211600	
H	-4.00674200	3.00692600	0.28262400	
H	4.27726700	1.94275900	-2.35245600	
H	-6.09640500	1.53502400	0.21398800	
H	0.08364900	-3.05434800	1.03042700	
H	-7.05689600	-0.74987300	0.00842800	
H	-0.37321000	4.69470600	1.37997100	
H	-0.05195200	2.97227500	1.69725300	
H	-1.58492000	3.71534900	2.24919000	
H	6.79781700	0.24466200	-0.54899200	
H	4.86237500	-1.22790900	2.71427500	
H	4.75892400	-2.57730000	1.54221400	
H	6.33310100	-2.11600700	2.24134500	
**Name**				**CMB-P**
Cartesian Coordinates				Energy
O	0.10107500	0.37070000	-0.35582100	Zero-point correction= 0.358205 (Hartree/Particle)
O	0.96068500	-2.26818100	0.15932800	Thermal correction to Energy= 0.382848
O	1.89313700	2.34247700	-0.45410700	Thermal correction to Enthalpy= 0.383793
O	-1.03446800	-3.63190700	-1.27824300	Thermal correction to Gibbs Free Energy= 0.302254
O	3.60728700	-2.81656100	0.40863500	Sum of electronic and zero-point Energies= -1374.247414
O	-4.45810200	1.68808800	1.47987200	Sum of electronic and thermal Energies= -1374.222770
O	-6.15688300	0.73366700	-0.29226200	Sum of electronic and thermal Enthalpies= -1374.221826
O	2.22072900	4.50772600	-0.71821100	Sum of electronic and thermal Free Energies= -1374.303365
C	-0.72878100	-0.75366200	-0.68227600	
C	-0.41734600	-1.88958200	0.29539500	
C	-2.16439300	-0.31624200	-0.60729500	
C	1.40752800	0.06133000	-0.17364300	
C	1.83783400	-1.23935900	0.07246800	
C	-1.20969200	-3.15756900	0.04236800	
C	2.35874000	1.08529700	-0.23355800	
C	3.21087000	-1.52434100	0.22211100	

C	3.71649200	0.81408200	-0.08023900
C	-2.58286600	0.52117400	0.43389500
C	-3.08593600	-0.79181600	-1.52964200
C	4.13516800	-0.50705900	0.14449000
C	-3.91898000	0.87455800	0.53289000
C	-4.43261500	-0.44484300	-1.42268400
C	4.62009500	1.93307400	-0.17190700
C	-4.85190200	0.38421900	-0.39795100
C	4.15395500	3.17611000	-0.38756200
C	2.72647500	3.43241600	-0.53441800
C	3.40559700	-3.29081600	1.74163800
C	-3.57307800	2.27720500	2.42289000
H	-0.48516500	-1.08653000	-1.69613700
H	-0.59031600	-1.53753800	1.32026000
H	-0.90194400	-3.90956100	0.77700900
H	-2.27302200	-2.95929500	0.17849100
H	-1.85651400	0.90564300	1.13887700
H	-2.75841400	-1.44158000	-2.33368700
H	5.18876300	-0.73552800	0.25691200
H	-5.16376200	-0.80513900	-2.13661700
H	5.68430200	1.75045300	-0.06365000
H	-0.10322800	-3.85511700	-1.38863000
H	4.79967400	4.04055600	-0.46112600
H	3.74214500	-4.32619300	1.75720000
H	2.34947300	-3.24558900	2.01740500
H	3.99950700	-2.70092400	2.44612900
H	-6.24957700	1.32837400	0.46384900
H	-4.18897500	2.90294300	3.06513500
H	-3.07999100	1.50918200	3.02491800
H	-2.82362300	2.89271000	1.91831100

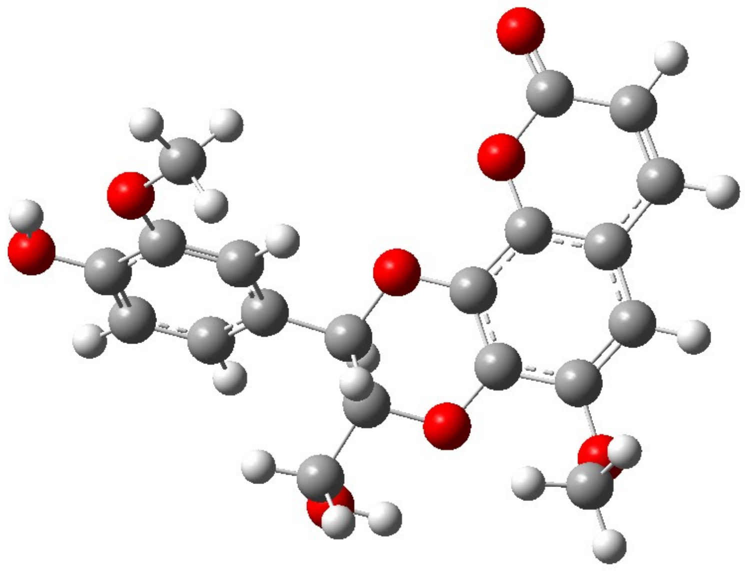

**Name**	**CMC-P**
Cartesian Coordinates	Energy

O	-0.12427600	-2.12104800	0.71019500
O	1.37177500	0.10588600	1.61349100
O	2.33886000	-1.02442400	3.99542000
O	3.23635600	1.15776400	0.04222900
O	0.34163800	-3.16360900	-1.75546100
O	-2.12323100	3.17544500	-0.10093300
O	-5.06439600	-0.49301500	-0.22387500
O	-4.45932300	2.14921900	-0.75927100
O	4.77052500	2.72739500	-0.17398900
C	-0.66484500	-1.24362300	1.72137800
C	0.48889700	-0.61282300	2.49254600
C	-1.65491500	-0.28339800	1.09610300
C	1.56901800	-0.45886000	0.39359900
C	0.82546500	-1.54327400	-0.06386500
C	1.30690000	-1.64343000	3.25710900
C	2.54865300	0.08880900	-0.44007300
C	1.06868100	-2.08664100	-1.34204000
C	-2.89613700	-0.79168100	0.72521000
C	-1.34774700	1.05515400	0.83141000
C	2.80258200	-0.44970500	-1.69929900
C	2.05128700	-1.54911600	-2.14304800
C	-2.28914900	1.86133800	0.20476900

Zero-point correction= 0.391050 (Hartree/Particle)
Thermal correction to Energy= 0.417458
Thermal correction to Enthalpy= 0.418402
Thermal correction to Gibbs Free Energy= 0.334685
Sum of electronic and zero-point Energies= -1488.719027
Sum of electronic and thermal Energies= -1488.692618
Sum of electronic and thermal Enthalpies= -1488.691674
Sum of electronic and thermal Free Energies= -1488.775392

C	-3.83725400	0.01522700	0.09303000
C	-3.54010200	1.35139800	-0.16736600
C	3.84014100	0.17605400	-2.47953000
C	4.51638000	1.23515100	-1.99925400
C	4.22505700	1.78292700	-0.68039200
C	-0.98610600	-2.82534500	-2.16572300
C	-0.88075300	3.78057500	0.23676600
C	-5.27024200	-0.67162100	-1.62612000
H	-1.19709100	-1.91475000	2.39671900
H	0.10641600	0.13749600	3.18672500
H	0.66111900	-2.15472200	3.97338900
H	1.71127200	-2.39049300	2.56266700
H	-3.15749100	-1.82602400	0.92363200
H	-0.39101000	1.46913500	1.11734900
H	2.23454100	-1.97887400	-3.12112300
H	4.06410400	-0.22511000	-3.46249400
H	2.87963300	-0.51787500	3.37878900
H	5.30043500	1.73194200	-2.55428000
H	-1.48314200	-3.76195600	-2.41393500
H	-0.95366100	-2.17993100	-3.04888500
H	-1.52864500	-2.32308200	-1.36096400
H	-4.07509000	3.03246300	-0.84546100
H	-0.71565200	3.75122300	1.31694100
H	-0.94984400	4.81447100	-0.09375500
H	-0.05434200	3.28446700	-0.27971300
H	-6.27510600	-1.07452700	-1.74324000
H	-4.54178300	-1.38366000	-2.02704800
H	-5.19296100	0.27821700	-2.15900200

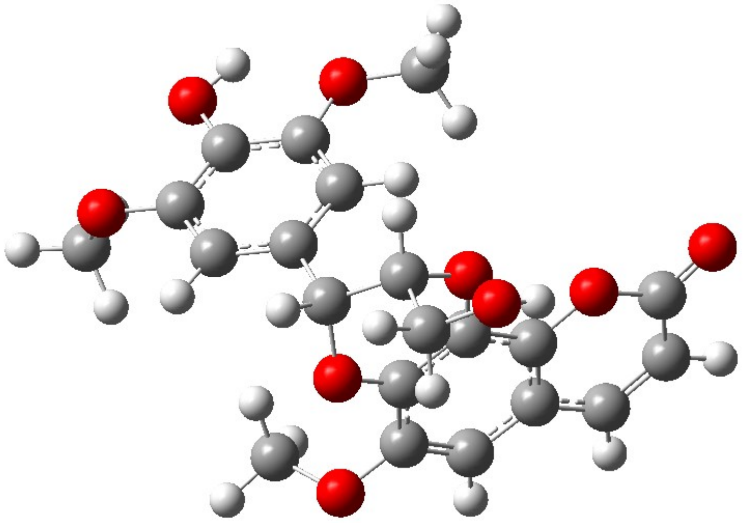

**Name**	**CMA-H**
Cartesian Coordinates	Energy

O	0.12543400	1.13834800	-0.11291400
O	-1.16903600	-1.37546600	-0.28016300
O	0.98010400	-2.94601400	0.80690700
O	-3.82745900	-1.49254200	-0.13741600
O	-1.32494700	3.40964300	0.07995700
O	5.68444900	-0.54318900	1.01414600
O	6.25732400	0.57934500	-1.33147800
O	-5.59577800	-2.80382600	-0.15987000
C	0.83935800	-0.09168100	0.12292200
C	0.19258100	-1.20144600	-0.70616700
C	2.27772200	0.12669800	-0.24984200
C	-1.85737400	-0.21169600	-0.13562300
C	-1.22405900	1.02427300	-0.05949800
C	0.86395800	-2.55033800	-0.55152500
C	-3.25037300	-0.25585000	-0.06686500
C	-1.98579100	2.20462700	0.05322700
C	-4.01055200	0.90424300	0.05189800
C	3.28365200	-0.30455800	0.62019100
C	2.61015400	0.69729000	-1.47288700
C	-3.35863400	2.14685100	0.10915600
C	4.61582600	-0.16210900	0.25850600
C	3.94798300	0.85229200	-1.83075600
C	-5.44020100	0.74930100	0.09564900
C	4.94675200	0.42427800	-0.97362200

Zero-point correction= 0.357311 (Hartree/Particle)
Thermal correction to Energy= 0.381993
Thermal correction to Enthalpy= 0.382937
Thermal correction to Gibbs Free Energy= 0.301757
Sum of electronic and zero-point Energies= -1374.251454
Sum of electronic and thermal Energies= -1374.226772
Sum of electronic and thermal Enthalpies= -1374.225828
Sum of electronic and thermal Free Energies= -1374.307008

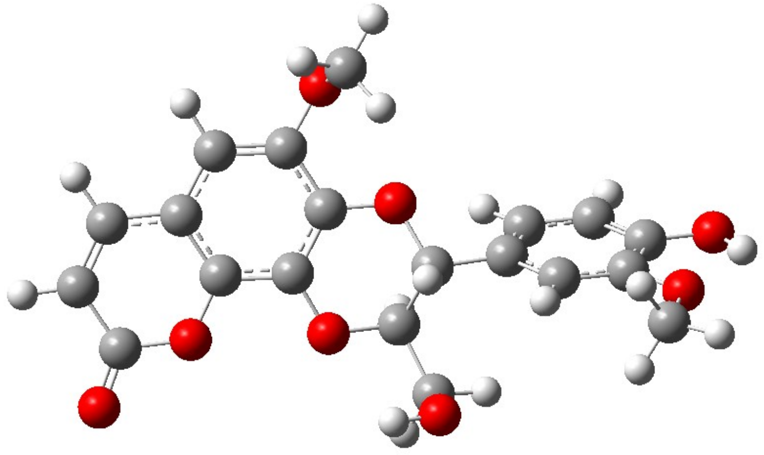

C	-6.00398800	-0.47249200	0.02503900
C	-5.18733500	-1.66325000	-0.09409000
C	-0.83835900	3.75100000	1.38681400
C	5.41011600	-1.15485100	2.27232900
H	0.74644300	-0.33788800	1.18440300
H	0.19522900	-0.90756900	-1.76224400
H	0.29490500	-3.28805600	-1.12430500
H	1.87336200	-2.49782300	-0.95998900
H	3.01505300	-0.74911100	1.57077700
H	1.83044600	1.02980700	-2.14848900
H	-3.93335600	3.06139100	0.19722200
H	4.22792000	1.30269900	-2.77582900
H	-6.05430600	1.63900300	0.18434200
H	0.09208700	-3.08599200	1.15707900
H	-7.07349400	-0.62827400	0.05402400
H	-0.30567800	4.69418900	1.28262200
H	-0.15851200	2.98012400	1.75620200
H	-1.67775300	3.87044600	2.07592500
H	6.81893200	0.23854800	-0.62099300
H	4.86838000	-0.46568800	2.92403700
H	4.83272600	-2.07195200	2.13485100
H	6.37827200	-1.38974800	2.70786800

**Name**	**CMB-H**
Cartesian Coordinates	Energy

Zero-point correction= 0.357338 (Hartree/Particle)
Thermal correction to Energy= 0.382005
Thermal correction to Enthalpy= 0.382950
Thermal correction to Gibbs Free Energy= 0.301866
Sum of electronic and zero-point Energies= -1374.251610
Sum of electronic and thermal Energies= -1374.226943
Sum of electronic and thermal Enthalpies= -1374.225999
Sum of electronic and thermal Free Energies= -1374.307082

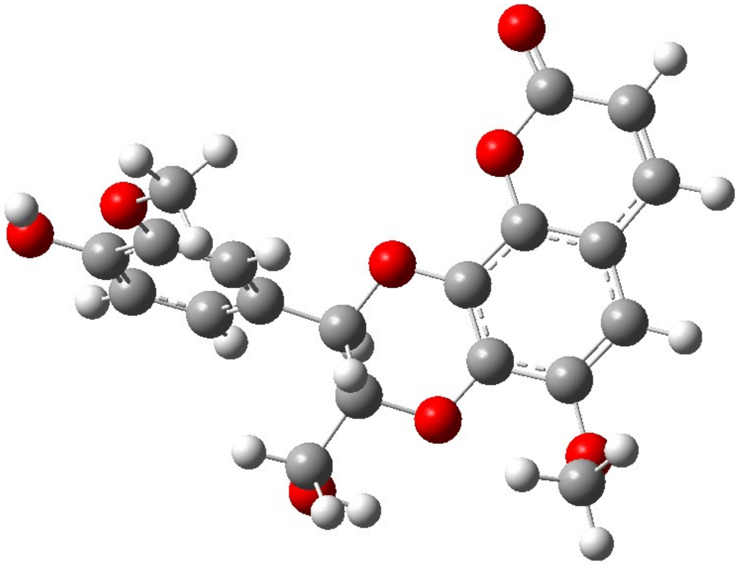

O	0.12785900	0.42400800	-0.38233700
O	0.91456100	-2.25944400	0.06719500
O	1.97772900	2.34120300	-0.41296100
O	-1.25464800	-3.49808400	-1.37631800
O	3.52705500	-2.89027800	0.34314400
O	-4.50772000	1.43387200	1.66617700
O	-6.12815000	0.94480500	-0.38200700
O	2.35806400	4.49840700	-0.63089000
C	-0.73043900	-0.67916900	-0.73619400
C	-0.45148600	-1.83722100	0.22194200
C	-2.15283300	-0.20669200	-0.66160900
C	1.42705800	0.06734300	-0.19331900
C	1.81755000	-1.25034600	0.01999700
C	-1.30973700	-3.05976600	-0.02748900
C	2.40804600	1.05998500	-0.21170500
C	3.18049800	-1.57007800	0.18028600
C	3.75559500	0.75586700	-0.04091100
C	-2.61241100	0.41875900	0.50468600
C	-3.02282000	-0.43873000	-1.71731900
C	4.13659600	-0.58203700	0.15254800
C	-3.93845700	0.81001400	0.59644200
C	-4.36063400	-0.05423600	-1.62124800
C	4.68525600	1.85308800	-0.08187900
C	-4.81787200	0.56524700	-0.47312700
C	4.25498900	3.11502700	-0.27633800
C	2.84584000	3.40198900	-0.45164200
C	3.39437600	-3.33980000	1.70012200
C	-3.66752400	1.71381600	2.78330700
H	-0.48355500	-0.98990700	-1.75501100
H	-0.59398800	-1.49360100	1.25312700

H	-0.98896800	-3.85349600	0.65315400	
H	-2.34981900	-2.81591900	0.19015900	
H	-1.92782100	0.59968500	1.32440400	
H	-2.66278400	-0.92177300	-2.61853500	
H	5.18143600	-0.83894600	0.28226300	
H	-5.05454600	-0.22606700	-2.43558300	
H	5.74122700	1.64118600	0.04744200	
H	-0.36382600	-3.82301400	-1.55266600	
H	4.92323600	3.96429100	-0.31134100	
H	3.65253400	-4.39693500	1.70313300	
H	2.36788700	-3.20792000	2.04929800	
H	4.08201100	-2.78607400	2.34374100	
H	-6.27375600	1.36078500	0.47959200	
H	-4.29942100	2.21059300	3.51546700	
H	-3.26908300	0.78827200	3.20506700	
H	-2.84831800	2.37460000	2.49096300	
**Name**				**CMC-H**
Cartesian Coordinates				Energy
O	-0.20490800	-1.75587200	1.30828100	Zero-point correction= 0.390372 (Hartree/Particle)
O	1.30624800	0.64195000	1.45369800	Thermal correction to Energy= 0.417699
O	2.28314500	0.25077000	4.08286600	Thermal correction to Enthalpy= 0.418644
O	3.21618800	1.09560800	-0.33497500	Thermal correction to Gibbs Free Energy= 0.330051
O	0.28808300	-3.55936500	-0.64638600	Sum of electronic and zero-point Energies= -1488.728082
O	-2.28230200	3.32121300	-0.74001500	Sum of electronic and thermal Energies= -1488.700755
O	-4.71898400	-0.63057200	-0.90487400	Sum of electronic and thermal Enthalpies= -1488.699810
O	-4.32607400	1.87279800	-1.64981000	Sum of electronic and thermal Free Energies= -1488.788403
O	4.78566200	2.48595800	-1.00540300	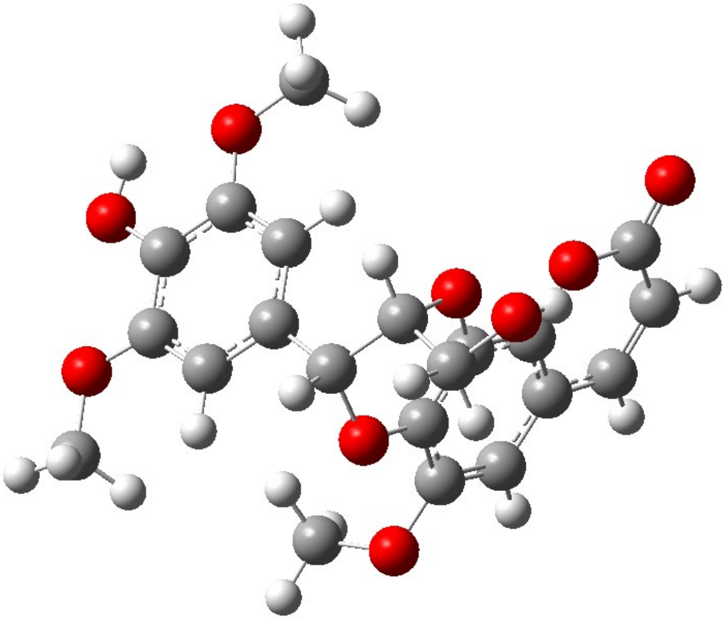
C	-0.73220400	-0.58461500	1.97288400	
C	0.43051100	0.23201600	2.52017000	
C	-1.66907300	0.14482500	1.02891600	
C	1.51452800	-0.29740900	0.49256800	
C	0.76986500	-1.46965200	0.40527100	
C	1.23093200	-0.54112200	3.55654500	
C	2.51373000	-0.07037700	-0.45540000	
C	1.03562700	-2.40603900	-0.61236700	
C	-2.75610700	-0.58499900	0.54401100	
C	-1.46807700	1.46279600	0.62899600	
C	2.78463600	-0.99133200	-1.46302100	
C	2.03039500	-2.17362100	-1.53339700	
C	-2.36569000	2.04439600	-0.26557800	
C	-3.64022200	-0.00389400	-0.35849900	
C	-3.44600500	1.31897700	-0.76348200	
C	3.84380500	-0.66545800	-2.38109400	
C	4.53213800	0.48587000	-2.25853500	
C	4.22668200	1.42671500	-1.19975800	
C	-0.95833000	-3.38655700	-1.33963300	
C	-1.17723500	4.11220000	-0.30773900	
C	-4.90987700	-2.00220600	-0.56639600	
H	-1.29649500	-0.98721900	2.81468000	
H	0.06336100	1.15897600	2.96158000	
H	0.57279800	-0.79898300	4.38723900	
H	1.62629000	-1.46578800	3.12302200	
H	-2.89627100	-1.60744500	0.87119400	
H	-0.62874700	2.03614200	0.99594000	

H	2.22504600	-2.90016800	-2.31356300	
H	4.07503400	-1.36815100	-3.17429800	
H	2.90020300	0.44109700	3.36605700	
H	5.33369300	0.76479100	-2.92837400	
H	-1.51429900	-4.31448000	-1.21875100	
H	-0.76998800	-3.19791300	-2.39944700	
H	-1.52112300	-2.55533000	-0.90874800	
H	-4.04185500	2.77878700	-1.83765100	
H	-1.20127600	4.24789100	0.77580300	
H	-1.28902400	5.07442800	-0.80154700	
H	-0.23354900	3.64988100	-0.60645400	
H	-5.78903900	-2.32477700	-1.11923100	
H	-5.08717700	-2.11626400	0.50553200	
H	-4.04421400	-2.59848900	-0.86679900	
**Name**				**CMA-anion**
Cartesian Coordinates				Energy
O	0.16898800	1.10195300	0.16610900	Zero-point correction= 0.344559 (Hartree/Particle)
O	-1.15329500	-1.32229400	-0.46247300	Thermal correction to Energy= 0.368748
O	0.95917700	-3.09724800	0.29009000	Thermal correction to Enthalpy= 0.369692
O	-3.80639800	-1.45775100	-0.26082600	Thermal correction to Gibbs Free Energy= 0.289524
O	-1.26008700	3.29841700	0.85205200	Sum of electronic and zero-point Energies= -1373.792021
O	5.70664800	-0.66594300	1.10557900	Sum of electronic and thermal Energies= -1373.767833
O	6.32176300	0.71557900	-1.10450500	Sum of electronic and thermal Enthalpies= -1373.766888
O	-5.58372900	-2.74062400	-0.46196700	Sum of electronic and thermal Free Energies= -1373.847056
C	0.88118400	-0.15992100	0.15462200	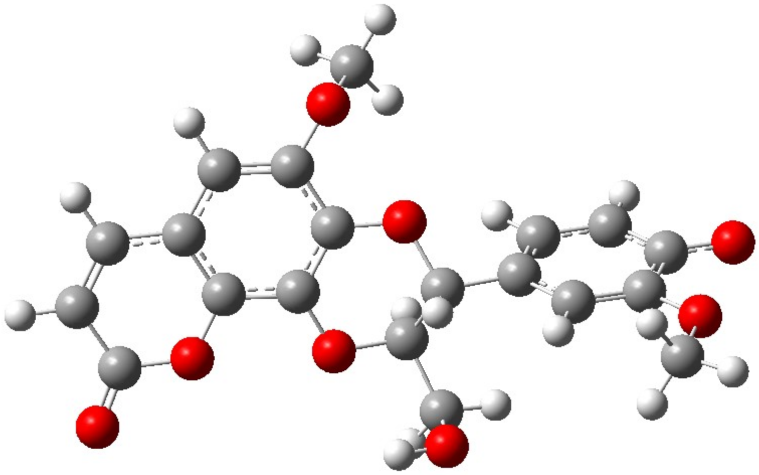
C	0.20931700	-1.08271400	-0.86043300	
C	2.31642800	0.11356100	-0.17155300	
C	-1.82670700	-0.20325100	-0.08628300	
C	-1.17969300	0.99318000	0.20636800	
C	0.86117200	-2.44555300	-0.96754600	
C	-3.21706800	-0.25691000	0.01962200	
C	-1.92993900	2.13467800	0.55847200	
C	-3.96318700	0.85865400	0.38885400	
C	3.32965200	-0.39446000	0.65708400	
C	2.67247200	0.81684600	-1.31669100	
C	-3.30053500	2.06950900	0.64821100	
C	4.66256600	-0.19869100	0.33993100	
C	4.01341800	1.02345100	-1.63141000	
C	-5.38958000	0.69520700	0.47145800	
C	5.06690500	0.52974400	-0.82885700	
C	-5.96505800	-0.49225800	0.19787000	
C	-5.16384200	-1.63463400	-0.19051400	
C	-0.81890700	4.00516500	-0.31710900	
C	5.37521900	-1.41616700	2.26388100	
H	0.78621500	-0.60143100	1.15079400	
H	0.20664100	-0.59741000	-1.84321300	
H	0.28695500	-3.05159000	-1.67438400	
H	1.87472300	-2.32950200	-1.35128200	
H	3.04912000	-0.94314300	1.54892500	
H	1.90003500	1.21592000	-1.96784300	
H	-3.86453600	2.95150300	0.92813300	
H	4.28438400	1.58237900	-2.52164700	
H	-5.99230400	1.55017900	0.75863900	
H	0.06460800	-3.25631100	0.61461700	

H	-7.03295800	-0.65317100	0.24918200
H	-0.28534400	4.88519700	0.03641700
H	-1.68229800	4.30749600	-0.91463000
H	-0.14979100	3.38269900	-0.91504500
H	4.80077900	-0.81328300	2.97285200
H	4.80566900	-2.31249800	2.00199600
H	6.32067700	-1.70636600	2.71814100

**Name**	**CMB-anion**
Cartesian Coordinates	Energy

Zero-point correction= 0.344500 (Hartree/Particle)
Thermal correction to Energy= 0.368736
Thermal correction to Enthalpy= 0.369680
Thermal correction to Gibbs Free Energy= 0.289075
Sum of electronic and zero-point Energies= -1373.792099
Sum of electronic and thermal Energies= -1373.767863
Sum of electronic and thermal Enthalpies= -1373.766918
Sum of electronic and thermal Free Energies= -1373.847524

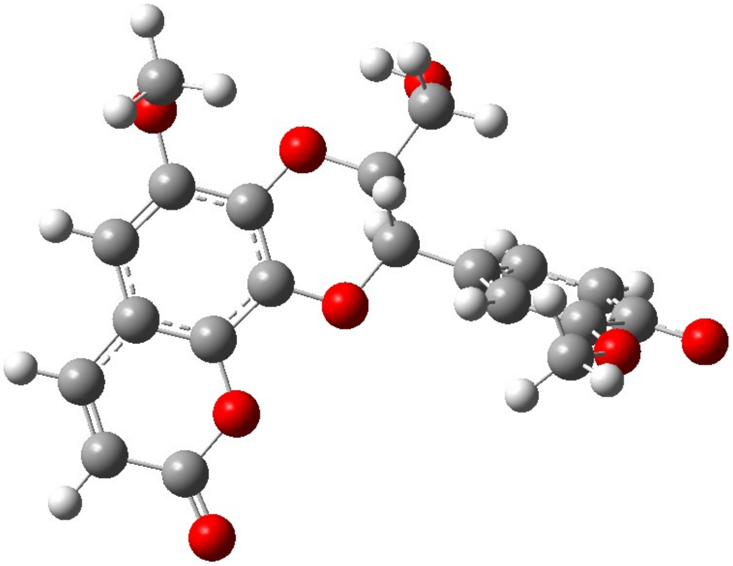

O	-0.11792700	-0.47028400	-0.32266200
O	-0.85477800	2.24102500	0.03836300
O	-2.01071700	-2.34912700	-0.32376700
O	1.31888800	3.39423400	-1.46330300
O	-3.45540800	2.93425200	0.25785700
O	4.61826100	-1.27817100	1.74997600
O	6.14599800	-1.09801300	-0.44300200
O	-2.43608100	-4.50240300	-0.49087200
C	0.76582900	0.60706700	-0.72300700
C	0.50742700	1.79879200	0.19698800
C	2.17675700	0.11528300	-0.66612400
C	-1.41104000	-0.08261900	-0.16089100
C	-1.77647500	1.24947200	0.00766800
C	1.38106900	2.99940900	-0.10073600
C	-2.41469000	-1.05287500	-0.16558300
C	-3.13471800	1.60262900	0.13739800
C	-3.75813200	-0.71489500	-0.02736500
C	2.71092900	-0.37524300	0.54059500
C	2.99712000	0.18855800	-1.78239600
C	-4.11251900	0.63585600	0.12197100
C	4.02991800	-0.78185200	0.60952600
C	4.33046500	-0.21377800	-1.71299800
C	-4.71145300	-1.79152600	-0.05713800
C	4.90914500	-0.71480000	-0.52715500
C	-4.30681100	-3.06766600	-0.21018500
C	-2.90171100	-3.39048600	-0.35059500
C	-3.32554700	3.42057600	1.60217100
C	3.80799700	-1.36121600	2.91246100
H	0.50301800	0.88842600	-1.74692800
H	0.65050400	1.49292800	1.23965900
H	1.07989800	3.82140700	0.55512800
H	2.41921000	2.74561600	0.11394200
H	2.07177900	-0.43685300	1.41400500
H	2.59622600	0.56780800	-2.71761600
H	-5.15332300	0.91924300	0.22650700
H	4.96429900	-0.15434600	-2.59210600
H	-5.76458300	-1.55273100	0.04591000
H	0.42984100	3.72250700	-1.64200400
H	-4.99357000	-3.90235900	-0.23646000
H	-3.56144800	4.48252800	1.57199300
H	-2.30539700	3.27776000	1.96517700
H	-4.03054600	2.90048200	2.25505000
H	4.44384300	-1.76718300	3.69686500
H	3.44642700	-0.37308000	3.21089800
H	2.95655400	-2.02838800	2.75162800

**Name**				**CMC-anion**
Cartesian Coordinates				Energy
O	-0.29165800	2.20128300	0.55365000	Zero-point correction= 0.377117 (Hartree/Particle)
O	1.47446000	1.04784900	-1.32042200	Thermal correction to Energy= 0.404118
O	2.33517600	3.43197400	-2.54814000	Thermal correction to Enthalpy= 0.405062
O	3.24977500	-0.75517200	-0.52294900	Thermal correction to Gibbs Free Energy= 0.318234
O	-0.22746400	1.41662900	3.15731500	Sum of electronic and zero-point Energies= -1488.268808
O	-1.58098000	-2.81780300	-1.61727800	Sum of electronic and thermal Energies= -1488.241807
O	-5.03545800	-0.03064700	0.10006800	Sum of electronic and thermal Enthalpies= -1488.240863
O	-4.08158900	-2.52370200	-0.67914500	Sum of electronic and thermal Free Energies= -1488.327692
O	4.87490400	-2.10456900	-1.14165700	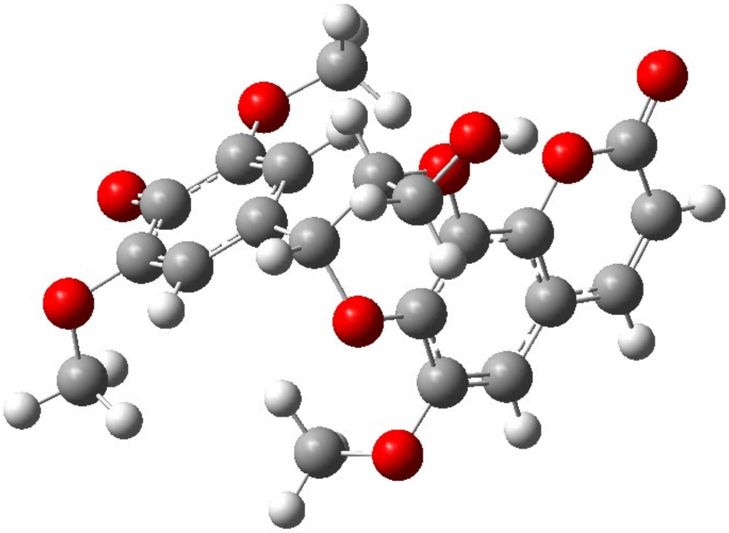
C	-0.72307500	2.03181900	-0.83136200	
C	0.51865300	2.05481800	-1.71552900	
C	-1.61311100	0.82276000	-0.93378700	
C	1.50198300	0.72745700	-0.00155100	
C	0.62510200	1.27346700	0.92947600	
C	1.20687500	3.40984200	-1.68792200	
C	2.42271300	-0.22826900	0.42966200	
C	0.67182000	0.86459600	2.27538500	
C	-2.91790800	0.93420500	-0.46171500	
C	-1.15205400	-0.44106800	-1.33689000	
C	2.49101300	-0.62281400	1.76322900	
C	1.59914600	-0.06308400	2.69225700	
C	-1.97755800	-1.55096600	-1.25325000	
C	-3.73877900	-0.18053100	-0.38176700	
C	-3.32034700	-1.48087700	-0.76295100	
C	3.47962900	-1.61197100	2.10274900	
C	4.29146700	-2.12817400	1.15958800	
C	4.19396400	-1.70118500	-0.22162900	
C	-1.52074200	0.79302200	3.08059000	
C	-0.22683200	-2.98255300	-2.01283900	
C	-5.11539900	-0.28686900	1.50366300	
H	-1.29897100	2.93540300	-1.03200000	
H	0.24723900	1.79839000	-2.74071400	
H	0.51084800	4.16682300	-2.05157500	
H	1.49849700	3.66156300	-0.66259300	
H	-3.30390400	1.89306800	-0.12643800	
H	-0.14668600	-0.55233000	-1.71983900	
H	1.62791300	-0.37082700	3.73088000	
H	3.55496400	-1.93465500	3.13563200	
H	2.97189100	2.78526600	-2.22080400	
H	5.04405400	-2.87341200	1.37673800	
H	-2.17619100	1.35246400	3.74563700	
H	-1.45033300	-0.24636500	3.41143000	
H	-1.90423000	0.83281300	2.05830200	
H	-0.01006000	-2.41866600	-2.92444800	
H	-0.09472500	-4.04555900	-2.20496900	
H	0.45551200	-2.66579900	-1.21725200	
H	-6.16351300	-0.19824400	1.78793900	
H	-4.52112000	0.44635700	2.05800800	
H	-4.75788100	-1.29477000	1.73208800	
**Name**				**CMA-O14-TS-G**
Cartesian Coordinates				Energy
O	-0.47593500	1.32416200	0.24417900	Zero-point correction= 0.370795 (Hartree/Particle)

O	-1.48954700	-1.24618900	-0.33514700
O	0.65608900	-2.73193900	0.75820100
O	-4.11305000	-1.66621400	-0.32830800
O	-2.21154500	3.39199500	0.72706800
O	5.08150900	-0.58506000	1.32656900
O	5.74944700	1.29602800	-0.41427400
O	-5.74071600	-3.14344000	-0.57288300
C	0.32338900	0.15246300	0.40802700
C	-0.13474900	-0.89761700	-0.61230000
C	1.76835500	0.52043000	0.22744800
C	-2.30669800	-0.18944300	-0.08533800
C	-1.81239000	1.08294700	0.18554300
C	0.64320100	-2.19926900	-0.55038200
C	-3.68869200	-0.40235000	-0.08139700
C	-2.69696600	2.15644800	0.42028200
C	-4.56760900	0.64862800	0.16685600
C	2.72741100	-0.21117600	0.92264500
C	2.14657100	1.52044800	-0.67905200
C	-4.05598700	1.93307800	0.40691800
C	4.07935400	0.04914000	0.72029500
C	3.48459200	1.77649600	-0.90118000
C	-5.97586800	0.33463400	0.15132500
C	4.48106600	1.05836900	-0.21283800
C	-6.39702700	-0.91735700	-0.09239700
C	-5.45192500	-2.00467800	-0.34980200
C	-1.55756400	4.06206400	-0.34693200
C	4.78440800	-1.78153300	2.02965300
H	0.15567100	-0.25125900	1.41186100
H	-0.06994700	-0.46074200	-1.61787200
H	0.19699400	-2.90115900	-1.26194400
H	1.68121500	-2.02436600	-0.83546000
H	2.40661000	-0.99219800	1.60171200
H	1.38303600	2.08506400	-1.19917300
H	-4.72215000	2.76401200	0.60530600
H	3.81264300	2.53678800	-1.59954600
H	-6.68698500	1.13190600	0.34108200
H	-0.24437100	-2.99108900	0.98004100
H	-7.44131200	-1.19588900	-0.11305300
H	-1.27190600	5.04059700	0.03311900
H	-2.24349500	4.18028900	-1.19109700
H	-0.66588100	3.51730600	-0.66192500
H	4.19081200	-1.57184700	2.92339900
H	4.24974500	-2.48008000	1.38045400
H	5.74424400	-2.20166700	2.31733000
O	5.24416400	-1.31494500	-1.41760100
H	5.02014700	-1.18388000	-2.35060800
O	6.48638200	-0.76108300	-1.29225000
H	6.25331800	0.34137100	-0.69232000

Thermal correction to Energy= 0.398146
Thermal correction to Enthalpy= 0.399090
Thermal correction to Gibbs Free Energy= 0.310922
Sum of electronic and zero-point Energies= -1525.082217
Sum of electronic and thermal Energies= -1525.054866
Sum of electronic and thermal Enthalpies= -1525.053921
Sum of electronic and thermal Free Energies= -1525.142090

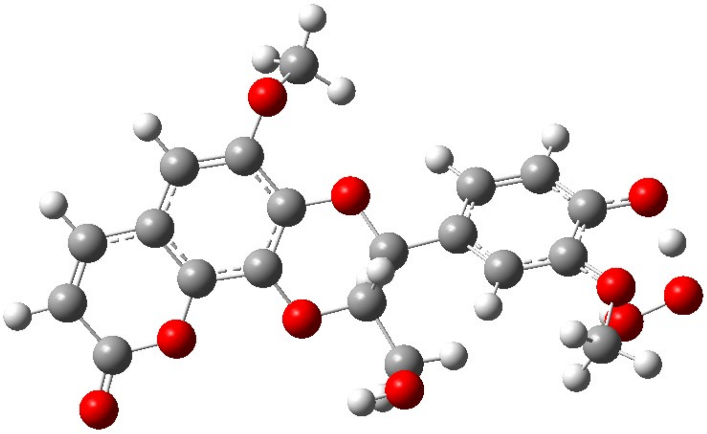

**Name**	**CMA-C18-TS-G**
Cartesian Coordinates	Energy

O	0.00530600	1.15269500	-0.45784100
O	-1.23747900	-1.37271900	-0.50609100
O	0.95226900	-2.89353300	0.44734800
O	-3.88413700	-1.54944100	-0.21639900

Zero-point correction= 0.370609 (Hartree/Particle)
Thermal correction to Energy= 0.398210
Thermal correction to Enthalpy= 0.399154
Thermal correction to Gibbs Free Energy= 0.311398

O	-1.48405300	3.40769300	-0.19985500	Sum of electronic and zero-point Energies= -1525.079859
O	5.46253800	-1.21019200	0.43217400	Sum of electronic and thermal Energies= -1525.052258
O	6.24746600	0.94479900	-0.87550600	Sum of electronic and thermal Enthalpies= -1525.051314
O	-5.64230300	-2.88670500	-0.10618900	Sum of electronic and thermal Free Energies= -1525.139070
C	0.73982900	-0.02089000	-0.28723000	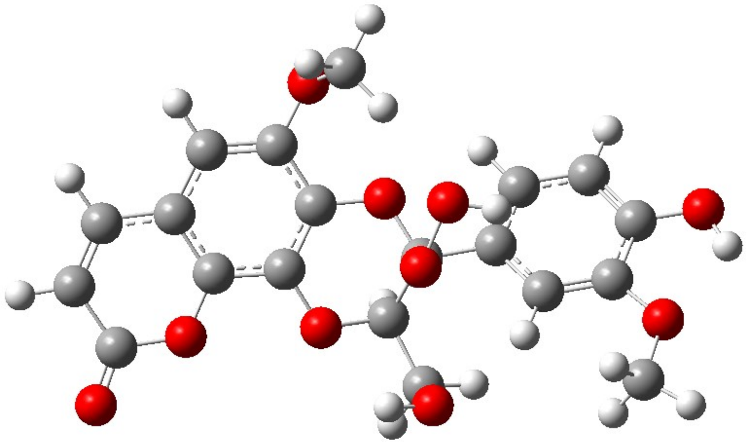
C	0.07784100	-1.17819500	-1.02984300	
C	2.18636500	0.24025800	-0.47898500	
C	-1.95128400	-0.22758700	-0.35114700	
C	-1.34376700	1.02196500	-0.33580400	
C	0.76556700	-2.52225100	-0.90095800	
C	-3.33848100	-0.30830100	-0.20392400	
C	-2.10530300	2.19680100	-0.18590000	
C	-4.10399200	0.84732600	-0.06187500	
C	3.11260600	-0.65773800	0.08381500	
C	2.64004300	1.37938200	-1.13899400	
C	-3.47314900	2.10124800	-0.05597800	
C	4.46665700	-0.42089500	-0.05676400	
C	4.00670100	1.61179500	-1.27620600	
C	-5.52947100	0.66743700	0.07210200	
C	4.92224300	0.72017600	-0.74508800	
C	-6.07010900	-0.56202900	0.06173900	
C	-5.24226800	-1.75985300	-0.08708200	
C	-0.76712200	3.70123200	1.00417200	
C	5.07882900	-2.38605100	1.12668000	
H	-0.00153900	-0.90072800	-2.09153000	
H	0.16100500	-3.26076200	-1.43734900	
H	1.75179200	-2.47814400	-1.36417200	
H	2.74593500	-1.51518500	0.63653800	
H	1.92483000	2.08271000	-1.54527800	
H	-4.05331900	3.00977200	0.05217300	
H	4.37973800	2.48634700	-1.79444000	
H	-6.15471500	1.54739200	0.18190300	
H	0.08921400	-2.93056100	0.87451700	
H	-7.13161300	-0.74132900	0.15987700	
H	-0.27967300	4.66006300	0.83981900	
H	-0.02220900	2.93243900	1.21689400	
H	-1.46642800	3.78118900	1.84145000	
H	6.72050900	0.22187000	-0.44478600	
H	4.50162500	-2.13703400	2.02120000	
H	4.48549000	-3.04078000	0.48244700	
H	6.00198800	-2.88447700	1.41100800	
O	1.24681500	1.07412000	2.37269100	
H	2.20376900	0.94185500	2.29259300	
O	0.71729200	-0.18492900	2.20700700	
H	0.64347400	-0.29191900	0.97206000	
**Name**				**CMB-O14-TS-G**
Cartesian Coordinates				Energy
O	0.47141700	0.17582600	-0.16761300	Zero-point correction= 0.370593 (Hartree/Particle)
O	1.87166700	-2.20676900	0.38997500	Thermal correction to Energy= 0.397984
O	1.78584500	2.47738700	-0.32276200	Thermal correction to Enthalpy= 0.398928
O	0.03592900	-4.00911500	-0.78227500	Thermal correction to Gibbs Free Energy= 0.310728
O	4.59619500	-2.15882000	0.63511500	Sum of electronic and zero-point Energies= -1525.081380
O	-4.15360800	1.07594600	1.44074800	Sum of electronic and thermal Energies= -1525.053989
O	-5.69472600	-0.65304400	0.15318800	Sum of electronic and thermal Enthalpies= -1525.053045

O	1.63587900	4.66699800	-0.59557900	Sum of electronic and thermal Free Energies= -1525.141245 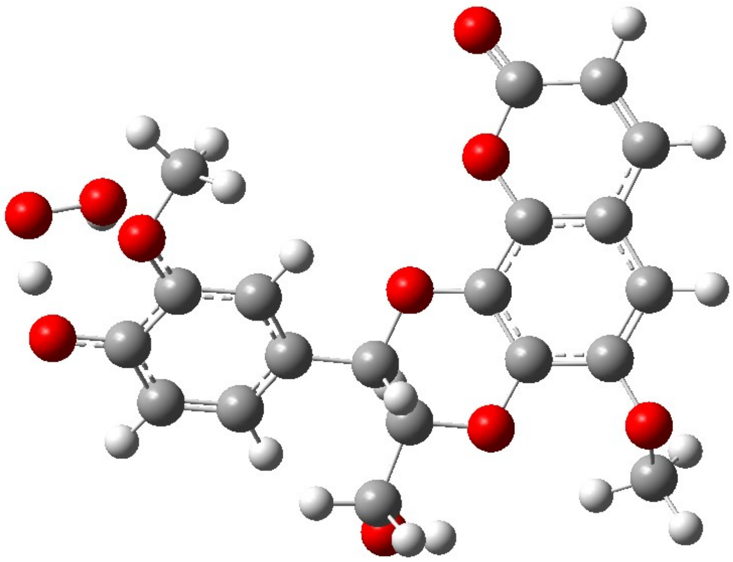
C	-0.12655700	-1.10064100	-0.38107700	
C	0.46561200	-2.08917600	0.63234400	
C	-1.61812400	-0.95274800	-0.24787900	
C	1.81912000	0.15147700	-0.02070700	
C	2.52078000	-1.02172800	0.23540000	
C	-0.08365200	-3.49886200	0.53013700	
C	2.52055000	1.35929900	-0.10541800	
C	3.92493600	-1.00402400	0.35691400	
C	3.90596200	1.38799800	0.03284400	
C	-2.15072200	0.05756000	0.54402900	
C	-2.45635800	-1.86665700	-0.90839600	
C	4.60286800	0.18990100	0.25324900	
C	-3.53290500	0.15839700	0.69949600	
C	-3.82439000	-1.76229000	-0.77997300	
C	4.54217000	2.67931200	-0.06947200	
C	-4.39921700	-0.75939500	0.02653300	
C	3.81426600	3.78762200	-0.28310700	
C	2.35777700	3.73082900	-0.41633000	
C	4.68037000	-3.06189800	-0.46175700	
C	-3.41622900	2.22100100	1.84142900	
H	0.12810800	-1.45118800	-1.38741500	
H	0.30833400	-1.69050200	1.64294200	
H	0.44634100	-4.12829600	1.25227500	
H	-1.14435900	-3.50163800	0.78033300	
H	-1.48547900	0.76900000	1.01375200	
H	-2.01593600	-2.64811200	-1.51882700	
H	5.68041700	0.19090200	0.36553900	
H	-4.49917400	-2.44043800	-1.28788600	
H	5.62115300	2.73516800	0.03057800	
H	0.97303600	-4.09138400	-0.98333400	
H	4.24949900	4.77375200	-0.36495300	
H	5.22806500	-3.93155400	-0.10468100	
H	5.22130800	-2.59974500	-1.29284500	
H	3.68493200	-3.36620800	-0.79394300	
H	-4.14003900	2.89667700	2.28899000	
H	-2.65428200	1.95752400	2.57984300	
H	-2.95216100	2.69372400	0.97245600	
O	-4.82757100	1.70307900	-1.19501500	
H	-4.83986900	1.49718100	-2.14123800	
O	-6.10679000	1.46749400	-0.78062300	
H	-6.00132900	0.38742800	-0.12241800	
**Name**				**CMB-C17-TS-G**
Cartesian Coordinates				Energy
O	-0.01503800	0.28975400	-0.07310800	Zero-point correction= 0.370307 (Hartree/Particle)
O	-1.19003100	-2.23209400	-0.48383400	Thermal correction to Energy= 0.397980
O	-1.54097700	2.44586500	0.14288200	Thermal correction to Enthalpy= 0.398924
O	0.87212600	-3.72712700	0.78746800	Thermal correction to Gibbs Free Energy= 0.310591
O	-3.91414400	-2.48027300	-0.49627900	Sum of electronic and zero-point Energies= -1525.078522
O	4.51911600	2.10062100	-0.96381300	Sum of electronic and thermal Energies= -1525.050849
O	6.21915300	0.33896000	0.01319300	Sum of electronic and thermal Enthalpies= -1525.049905
O	-1.59474200	4.63819000	0.42108600	Sum of electronic and thermal Free Energies= -1525.138238
C	0.72748000	-0.87745800	0.10961500	
C	0.19441000	-2.00445600	-0.77592700	

C	2.17957600	-0.56832500	0.07119100	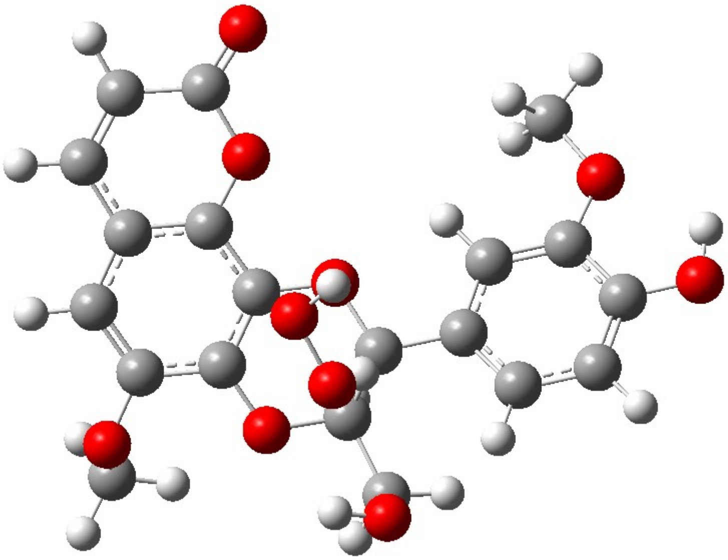
C	-1.36266700	0.12918900	-0.16382100	
C	-1.94672700	-1.11545900	-0.34739900	
C	0.86004300	-3.35076800	-0.56948600	
C	-2.17179200	1.25835200	-0.02871000	
C	-3.34968500	-1.24495700	-0.37116700	
C	-3.55869400	1.14081600	-0.05105600	
C	2.61433700	0.65654600	-0.45967600	
C	3.10981900	-1.46139600	0.60391700	
C	-4.13882600	-0.12626900	-0.22149000	
C	3.96479200	0.95349000	-0.47889400	
C	4.46650500	-1.15744600	0.57775300	
C	-4.31386600	2.35757900	0.11844800	
C	4.90159000	0.04054300	0.03547400	
C	-3.68976900	3.53646300	0.28262700	
C	-2.23043100	3.63202000	0.29396400	
C	-3.83218100	-3.02968300	-1.80806400	
C	3.63759900	3.10559400	-1.43517800	
H	0.28430000	-1.69026500	-1.82628900	
H	0.32580300	-4.08364000	-1.18470200	
H	1.89661500	-3.30864400	-0.90441900	
H	1.88412100	1.35862300	-0.83721300	
H	2.76827600	-2.37989500	1.06764500	
H	-5.21600400	-0.24256200	-0.23004900	
H	5.20289100	-1.83477500	0.99167500	
H	-5.39745600	2.30086900	0.11329300	
H	-0.02178900	-3.64730600	1.14246700	
H	-4.21733900	4.47088800	0.41286100	
H	-4.32079000	-4.00095300	-1.76518300	
H	-2.79144100	-3.15558400	-2.11407400	
H	-4.35515400	-2.38502200	-2.52091200	
H	6.32171800	1.21624200	-0.37631600	
H	4.26474700	3.93918100	-1.74104600	
H	3.06251300	2.74483200	-2.29231500	
H	2.95533800	3.42681000	-0.64373600	
O	-0.71345500	-0.28153900	2.71992700	
H	-0.04749200	0.23861400	3.19068800	
O	-0.06790400	-1.46121300	2.42258900	
H	0.43921900	-1.26281300	1.30400800	
**Name**				**CMC-O14-TS-G**
Cartesian Coordinates				Energy
O	-0.28946400	-2.43923200	0.54786700	Zero-point correction= 0.403434 (Hartree/Particle)
O	-1.73576300	-0.96806600	-1.37375400	Thermal correction to Energy= 0.433551
O	-3.01577600	-3.12927700	-2.61364900	Thermal correction to Enthalpy= 0.434495
O	-3.24767900	1.07073300	-0.62603700	Thermal correction to Gibbs Free Energy= 0.339554
O	-0.45983700	-1.70279500	3.23137200	Sum of electronic and zero-point Energies= -1639.554968
O	1.95168000	2.16423600	-1.91419100	Sum of electronic and thermal Energies= -1639.524852
O	4.53763100	-0.89252900	0.64601800	Sum of electronic and thermal Enthalpies= -1639.523907
O	4.11366100	1.68046200	-0.50559400	Sum of electronic and thermal Free Energies= -1639.618848
O	-4.61295800	2.68423800	-1.27568500	
C	0.19444000	-2.33995900	-0.80341800	
C	-0.99884300	-2.14563000	-1.72948200	
C	1.26939400	-1.27135100	-0.86685100	
C	-1.78290200	-0.66951100	-0.04743100	

C	-1.05974000	-1.37531500	0.91093700
C	-1.95561700	-3.33039400	-1.71167800
C	-2.58133400	0.40458100	0.35197900
C	-1.13060900	-1.01456700	2.27077400
C	2.46027600	-1.53889200	-0.19148900
C	1.06337200	-0.04154800	-1.49057800
C	-2.67816000	0.75208100	1.69702500
C	-1.94646700	0.03225300	2.65092300
C	2.04798500	0.93600000	-1.40100900
C	3.44930300	-0.57231100	-0.07982000
C	3.25066800	0.70626800	-0.66568500
C	-3.53912600	1.86540300	2.01902600
C	-4.19902800	2.52405000	1.05252600
C	-4.07120000	2.14533200	-0.35566500
C	0.94256100	-1.89596700	3.04383200
C	0.67438400	2.60257200	-2.35456300
C	5.80923200	-0.31463400	0.33492700
H	0.63789300	-3.31694600	-1.00428700
H	-0.66130300	-1.97096600	-2.75348400
H	-1.42807900	-4.23049900	-2.03429100
H	-2.31912600	-3.49196900	-0.68904800
H	2.62681100	-2.49808600	0.28526600
H	0.13798200	0.16822800	-2.00659000
H	-2.00431200	0.29028800	3.70129300
H	-3.64114500	2.15809300	3.05884900
H	-3.44042800	-2.29359600	-2.39248800
H	-4.85256100	3.36235200	1.24932900
H	1.38299100	-1.91699400	4.03949300
H	1.37412500	-1.07416100	2.46475500
H	1.13797800	-2.83561300	2.52835300
H	0.35741800	2.05481500	-3.24569700
H	0.78989000	3.65586700	-2.59490200
H	-0.06306100	2.47986700	-1.55688700
H	5.93149800	0.64807900	0.82601300
H	5.91914300	-0.18572700	-0.74289600
H	6.54535200	-1.02724200	0.70231200
O	1.75326000	2.41842400	0.91363000
H	1.81785000	2.04021600	1.80352500
O	2.86072000	3.20119500	0.79344500
H	3.59576400	2.53410700	-0.03168000

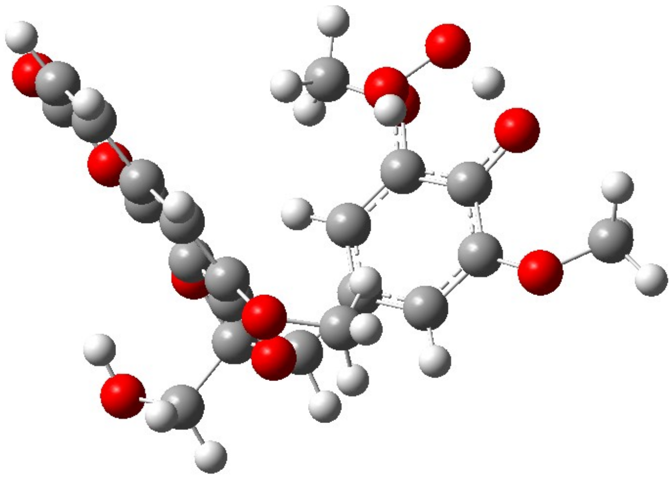

**Name**	**CMC-C18-TS-G**
Cartesian Coordinates	Energy

O	0.20490400	-0.02529900	-1.68920300	Zero-point correction= 0.402505 (Hartree/Particle)
O	1.15190500	-1.33908500	0.61260000	Thermal correction to Energy= 0.433193
O	1.71495200	-3.86440600	-0.49043900	Thermal correction to Enthalpy= 0.434137
O	3.51131900	-0.65851100	1.65403200	Thermal correction to Gibbs Free Energy= 0.337807
O	1.76613700	1.90270700	-2.90066800	Sum of electronic and zero-point Energies= -1639.544732
O	-4.76624000	-1.11209700	1.63933300	Sum of electronic and thermal Energies= -1639.514044
O	-2.88707500	3.07852800	0.58795700	Sum of electronic and thermal Enthalpies= -1639.513099
O	-4.85887300	1.50958100	1.76668800	Sum of electronic and thermal Free Energies= -1639.609429
O	5.00610200	-1.03539000	3.24021500	
C	-0.59872300	-0.92811500	-0.98308600	
C	0.22282200	-1.98518300	-0.27119600	
C	-1.68731100	-0.27147500	-0.22535200	

C	1.86138900	-0.34324400	0.01684300	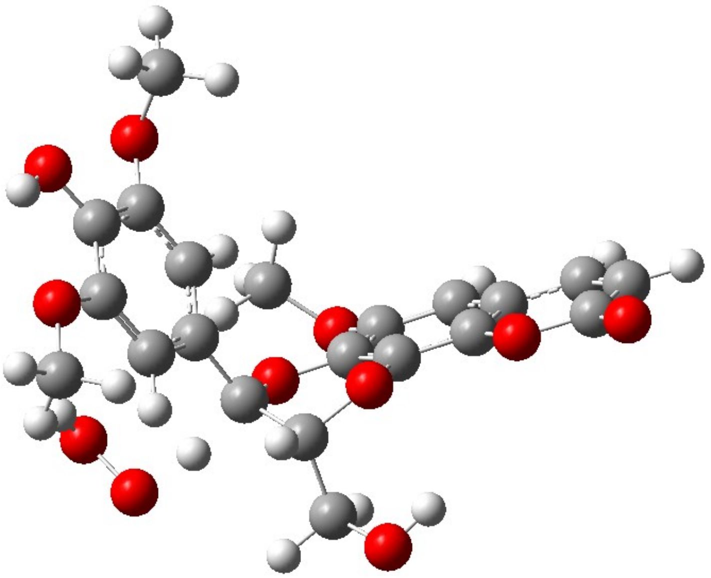
C	1.39921700	0.30851100	-1.12245300	
C	0.98154200	-2.90832700	-1.21673800	
C	3.09757800	0.02140100	0.55521200	
C	2.16585600	1.31647700	-1.74064300	
C	-1.77289500	1.11370000	-0.13540300	
C	-2.68491600	-1.07219800	0.36189600	
C	3.85944300	1.02576900	-0.03763600	
C	3.38090500	1.66635100	-1.18923800	
C	-3.73136600	-0.46788500	1.03431400	
C	-2.82274900	1.71984600	0.55035700	
C	-3.81531600	0.93354200	1.13150700	
C	5.12398700	1.33469900	0.58581300	
C	5.52878500	0.67052700	1.68067800	
C	4.71518800	-0.39054200	2.27649100	
C	0.53233700	2.62133100	-2.86775000	
C	-4.76774600	-2.52938500	1.61184400	
C	-2.78445500	3.65597800	1.88977800	
H	-0.40918900	-2.58266900	0.38469500	
H	0.26545500	-3.44980400	-1.83821600	
H	1.62944600	-2.32147300	-1.88060200	
H	-1.02640900	1.75092200	-0.59025800	
H	-2.63801900	-2.15007600	0.26874100	
H	3.96489300	2.43987500	-1.67297300	
H	5.74049400	2.11385600	0.14978100	
H	2.26889600	-3.40071000	0.14718600	
H	6.46753300	0.86912100	2.17855400	
H	0.52563000	3.24399700	-3.75997700	
H	0.49055500	3.26057400	-1.98024500	
H	-0.32043400	1.94251400	-2.88580800	
H	-5.43591900	0.80715100	2.09295300	
H	-4.81585400	-2.89713100	0.58309600	
H	-5.65707600	-2.84154400	2.15307900	
H	-3.87688100	-2.92539700	2.10643800	
H	-2.79221200	4.73368600	1.74194900	
H	-1.84290400	3.35615400	2.35880800	
H	-3.62621200	3.36229900	2.51739400	
O	-2.89917700	-0.91732900	-2.92331400	
H	-3.55092300	-0.92070000	-2.20526300	
O	-2.10957300	-2.01033400	-2.67134500	
H	-1.25736500	-1.56821800	-1.90761500	

**Name**				**CMA-O14-TS-P**
Cartesian Coordinates				Energy
O	-0.48137300	1.30373300	-0.04022700	Zero-point correction= 0.370117 (Hartree/Particle)
O	-1.53278800	-1.31329600	-0.18761900	Thermal correction to Energy= 0.397510
O	0.63787500	-2.64209800	1.08797100	Thermal correction to Enthalpy= 0.398455
O	-4.17315700	-1.67477600	-0.18614500	Thermal correction to Gibbs Free Energy= 0.309994
O	-2.15566200	3.44383800	-0.02531100	Sum of electronic and zero-point Energies= -1525.120104
O	5.09168300	-0.32852800	1.30571900	Sum of electronic and thermal Energies= -1525.092710
O	5.71318300	1.23759500	-0.77360100	Sum of electronic and thermal Enthalpies= -1525.091766
O	-5.81970000	-3.14097000	-0.24913300	Sum of electronic and thermal Free Energies= -1525.180227
C	0.31118100	0.15840500	0.28979200	
C	-0.17474400	-1.03456800	-0.54216300	
C	1.75208000	0.48574600	0.01866300	

C	-2.33225000	-0.21844200	-0.12670300
C	-1.81762700	1.07295700	-0.06090900
C	0.59426100	-2.31621000	-0.28923600
C	-3.71895500	-0.39565400	-0.13478400
C	-2.68310600	2.18664500	-0.03419000
C	-4.58080400	0.69774100	-0.10283500
C	2.72667600	-0.05083800	0.85709800
C	2.11001800	1.25019900	-1.10088300
C	-4.04651500	1.99537000	-0.05635200
C	4.07194600	0.16859800	0.58203900
C	3.44132600	1.47405500	-1.38104100
C	-5.99396900	0.41477200	-0.12926400
C	4.45331700	0.95846100	-0.54799100
C	-6.43956300	-0.85329700	-0.17864400
C	-5.51398100	-1.97896400	-0.20788600
C	-1.64674000	3.84653200	1.24875900
C	4.77677200	-1.05718100	2.49335500
H	0.16678100	-0.07554900	1.34944000
H	-0.12569000	-0.77138900	-1.60637200
H	0.13121800	-3.11806500	-0.87283000
H	1.62409200	-2.19822100	-0.62837000
H	2.42253700	-0.64749100	1.70827800
H	1.33786200	1.66682200	-1.73605200
H	-4.70405800	2.85687500	-0.03982800
H	3.74901900	2.06502600	-2.23559600
H	-6.69021400	1.24693400	-0.10981200
H	-0.25658400	-2.86933800	1.36637600
H	-7.49098900	-1.10550400	-0.20062100
H	-1.25098900	4.85263000	1.12021400
H	-0.84733800	3.17990200	1.57973000
H	-2.45222900	3.86010500	1.98910300
H	4.22060400	-0.42819200	3.19184000
H	4.20050800	-1.95632400	2.25864000
H	5.73280900	-1.33590100	2.92988800
O	5.59840300	-1.61974800	-1.20103100
H	5.50212700	-1.78842300	-0.24788600
O	6.70775200	-0.83602100	-1.28850600
H	6.30485700	0.30232100	-0.93403600

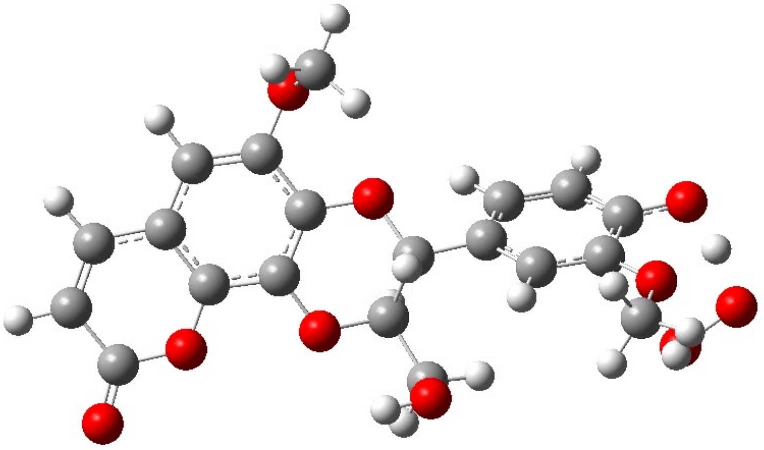

Name	CMA-C18-TS-P
Cartesian Coordinates	Energy

O	0.00264300	1.14736200	-0.42600000
O	-1.24297500	-1.37485500	-0.49083900
O	0.95020800	-2.92343800	0.43506600
O	-3.89842800	-1.54339400	-0.22066600
O	-1.48220500	3.40401900	-0.19813700
O	5.47883100	-1.18488600	0.40536600
O	6.23659700	0.96851600	-0.91210700
O	-5.65185300	-2.87765000	-0.12460900
C	0.74019600	-0.02808700	-0.26761000
C	0.07650900	-1.18065500	-1.01731300
C	2.18445900	0.23983700	-0.47388500
C	-1.95540400	-0.23096200	-0.33999700
C	-1.34606600	1.01817000	-0.31846100
C	0.76326500	-2.52520400	-0.90821800

Zero-point correction= 0.369658 (Hartree/Particle)
Thermal correction to Energy= 0.397448
Thermal correction to Enthalpy= 0.398392
Thermal correction to Gibbs Free Energy= 0.310043
Sum of electronic and zero-point Energies= -1525.113867
Sum of electronic and thermal Energies= -1525.086077
Sum of electronic and thermal Enthalpies= -1525.085133
Sum of electronic and thermal Free Energies= -1525.173481

C	-3.34395800	-0.30377300	-0.20279200	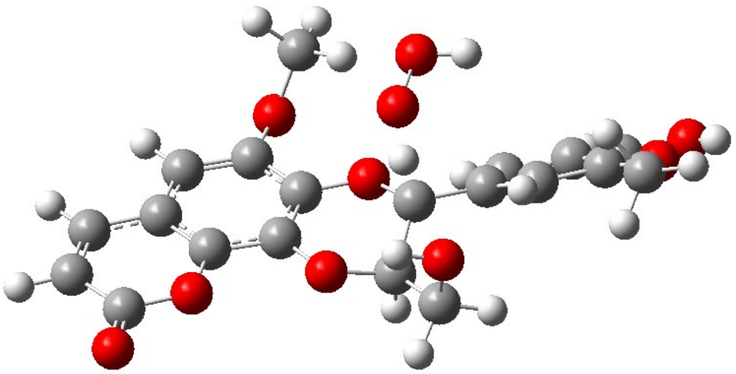
C	-2.10786800	2.19496300	-0.17879500	
C	-4.10928500	0.85214700	-0.06464400	
C	3.12172900	-0.65350600	0.07782100	
C	2.62417300	1.37992800	-1.14252400	
C	-3.47664700	2.10549000	-0.05550100	
C	4.47394500	-0.40831900	-0.07406300	
C	3.98813500	1.62017900	-1.29543700	
C	-5.53532900	0.67787500	0.05870600	
C	4.91328600	0.73574500	-0.76941000	
C	-6.08003500	-0.55145500	0.04307500	
C	-5.25311700	-1.74389900	-0.10082600	
C	-0.78411300	3.70961700	1.01604200	
C	5.11605200	-2.36058700	1.12015100	
H	-0.00376900	-0.89451300	-2.07582500	
H	0.16145200	-3.25535500	-1.45887100	
H	1.74745700	-2.47423600	-1.37476600	
H	2.77015200	-1.51792100	0.62909400	
H	1.90539500	2.07956000	-1.54923900	
H	-4.06051000	3.01242200	0.05003700	
H	4.34516700	2.49728300	-1.82213700	
H	-6.15800300	1.56014800	0.16467000	
H	0.08355500	-3.01236200	0.84864300	
H	-7.14409200	-0.72288300	0.13358300	
H	-0.30493800	4.67476400	0.85967100	
H	-0.02881000	2.95243800	1.23605600	
H	-1.49306600	3.78087900	1.84615800	
H	6.72076700	0.25021300	-0.48255100	
H	4.54681200	-2.10736200	2.01864900	
H	4.53027000	-3.03275600	0.48730400	
H	6.04941100	-2.84271300	1.40182100	
O	1.30687200	1.00914200	2.43481300	
H	2.25922400	0.83548600	2.35608900	
O	0.72030700	-0.22020000	2.23528500	
H	0.65398900	-0.30504400	0.98833000	
**Name**				**CMB-O14-TS-P**
Cartesian Coordinates				Energy
O	0.48448800	0.20199300	-0.22717000	Zero-point correction= 0.370143 (Hartree/Particle)
O	1.84815000	-2.24294500	0.10423400	Thermal correction to Energy= 0.397478
O	1.86703500	2.47908800	-0.33024900	Thermal correction to Enthalpy= 0.398422
O	-0.01388700	-3.91086800	-1.22571200	Thermal correction to Gibbs Free Energy= 0.310244
O	4.56416100	-2.29561400	0.14659700	Sum of electronic and zero-point Energies= -1525.119507
O	-4.10546900	1.00457000	1.54288800	Sum of electronic and thermal Energies= -1525.092172
O	-5.69059700	-0.64617700	0.16337600	Sum of electronic and thermal Enthalpies= -1525.091228
O	1.76695000	4.67591700	-0.49866200	Sum of electronic and thermal Free Energies= -1525.179406
C	-0.13780800	-1.04276600	-0.55435300	
C	0.44182200	-2.13008800	0.36005700	
C	-1.62423200	-0.88224400	-0.39050400	
C	1.83700700	0.13841600	-0.14129900	
C	2.51508000	-1.06660200	0.01090000	
C	-0.12497900	-3.51659800	0.12765300	
C	2.57300700	1.32526400	-0.20802500	
C	3.92417200	-1.09469200	0.05428100	
C	3.96478400	1.30813900	-0.15789800	

C	-2.13129300	0.02333800	0.53663800
C	-2.48643600	-1.67793500	-1.16131900
C	4.63546700	0.08103100	-0.03120400
C	-3.50848100	0.13729200	0.70626200
C	-3.85019400	-1.57420700	-0.99773200
C	4.63628600	2.58043300	-0.24966200
C	-4.39814900	-0.68674700	-0.05028200
C	3.93336600	3.72086700	-0.36813100
C	2.47624200	3.70964600	-0.40680200
C	4.57255500	-2.84002800	1.46827900
C	-3.27532800	1.82153400	2.36945300
H	0.09964200	-1.29639800	-1.59306700
H	0.28993800	-1.83076000	1.40480000
H	0.39601200	-4.21612000	0.78974800
H	-1.18433800	-3.53032500	0.38488300
H	-1.45032500	0.64516200	1.10119400
H	-2.06930600	-2.37347400	-1.88150000
H	5.71856600	0.05099600	-0.00072500
H	-4.53850500	-2.17672800	-1.57849100
H	5.72096900	2.59739900	-0.22165200
H	0.92362300	-3.98146300	-1.43885700
H	4.40078000	4.69349400	-0.43894900
H	5.09677000	-3.79251100	1.40867600
H	3.55478300	-3.00478000	1.82982600
H	5.10489700	-2.16951900	2.14947200
H	-3.95634900	2.42257400	2.96728800
H	-2.65729600	1.20267900	3.02413500
H	-2.64071500	2.47364100	1.76319400
O	-5.12607900	1.92643300	-1.00699800
H	-4.65681100	2.25387700	-0.22063200
O	-6.33220500	1.51423000	-0.53134900
H	-6.09004000	0.37492900	-0.04286200

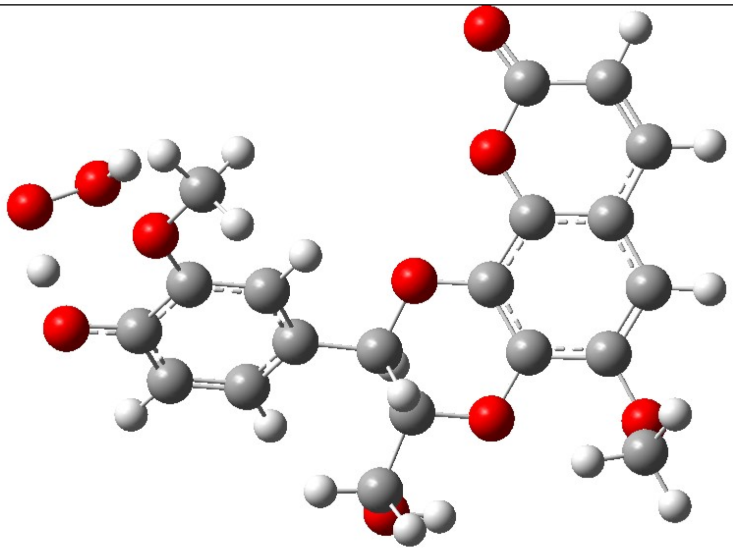

**Name**	**CMB-C17-TS-P**
Cartesian Coordinates	Energy

O	-0.02755200	0.29499500	-0.04261900	Zero-point correction= 0.369493 (Hartree/Particle)
O	-1.17444800	-2.23851400	-0.46429400	Thermal correction to Energy= 0.397294
O	-1.58972700	2.43613800	0.16416300	Thermal correction to Enthalpy= 0.398238
O	0.88843600	-3.74406400	0.77152300	Thermal correction to Gibbs Free Energy= 0.309476
O	-3.88228100	-2.52155600	-0.51447900	Sum of electronic and zero-point Energies= -1525.112911
O	4.51529100	2.08976500	-1.03436500	Sum of electronic and thermal Energies= -1525.085111
O	6.21599100	0.37262100	0.00853300	Sum of electronic and thermal Enthalpies= -1525.084167
O	-1.66911800	4.62150000	0.44868600	Sum of electronic and thermal Free Energies= -1525.172929
C	0.72738700	-0.86609500	0.12945800	
C	0.20768900	-1.99344100	-0.76364100	
C	2.17795300	-0.54279200	0.08748900	
C	-1.37287200	0.11962200	-0.14176800	
C	-1.94216700	-1.13163300	-0.33399100	
C	0.89202100	-3.33169400	-0.57939700	
C	-2.19961900	1.23732500	-0.01514400	
C	-3.34393800	-1.27675600	-0.37445800	
C	-3.58496100	1.10445900	-0.05523200	
C	2.61082500	0.66403800	-0.48487600	
C	3.10941400	-1.41718800	0.64724000	
C	-4.14985000	-0.16880200	-0.23494700	

C	3.96194500	0.96427900	-0.51094300
C	4.46635000	-1.11154400	0.61668700
C	-4.35847000	2.30971100	0.10555600
C	4.89841800	0.07039600	0.03827200
C	-3.75110100	3.49745200	0.27924200
C	-2.29811500	3.60620200	0.30944200
C	-3.82924200	-3.02259800	-1.85202000
C	3.63483000	3.07835800	-1.55417500
H	0.28985300	-1.67054300	-1.81118300
H	0.38186800	-4.05918100	-1.22010100
H	1.93156000	-3.26628100	-0.89994400
H	1.88559300	1.35152000	-0.89837700
H	2.77238300	-2.32838400	1.12824400
H	-5.22665900	-0.28963600	-0.26123100
H	5.19975300	-1.77931900	1.05283900
H	-5.44092500	2.23855200	0.08597000
H	-0.02231500	-3.73616200	1.09092700
H	-4.29709900	4.42262900	0.40395300
H	-4.26639400	-4.01947100	-1.82773100
H	-2.79763200	-3.08552000	-2.20609300
H	-4.41304300	-2.38106100	-2.51883200
H	6.31919900	1.23743800	-0.41117500
H	4.26633000	3.89758700	-1.89065800
H	3.06557800	2.68390900	-2.39995900
H	2.95137500	3.43478200	-0.77907200
O	-0.57569200	-0.20448200	2.80007700
H	0.14501000	0.31387900	3.19036900
O	0.00707600	-1.41236500	2.48376200
H	0.45276900	-1.25284600	1.32741000

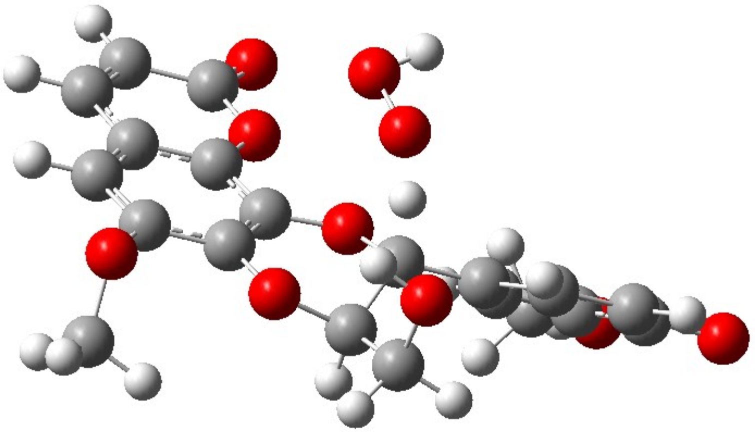

**Name**	**CMC-O14-TS-P**
Cartesian Coordinates	Energy

O	-0.41261200	-2.31651300	0.66233800
O	-1.80660900	-0.91313900	-1.36328300
O	-3.13938400	-3.10018500	-2.50748600
O	-3.39724100	1.10739900	-0.70270900
O	-0.65853700	-1.55608200	3.25792300
O	2.07172400	2.09234700	-1.77701700
O	4.66980600	-1.30927000	0.28823200
O	4.43852900	1.21878200	-0.96023000
O	-4.77569900	2.66340100	-1.43956900
C	0.07387500	-2.32828700	-0.69353100
C	-1.09709900	-2.13030500	-1.64866600
C	1.22111600	-1.34391100	-0.81583000
C	-1.91418500	-0.59138600	-0.04736700
C	-1.21330000	-1.26049600	0.95134100
C	-2.08371100	-3.28825000	-1.59045100
C	-2.74468100	0.47564000	0.30841400
C	-1.34418800	-0.87094200	2.30003800
C	2.44280800	-1.73381400	-0.27340000
C	1.05093100	-0.05976400	-1.34664100
C	-2.89156300	0.85710200	1.63976500
C	-2.18054500	0.17024800	2.63644000
C	2.12230700	0.81988300	-1.33789700
C	3.52023900	-0.85759600	-0.24018900

Zero-point correction= 0.403160 (Hartree/Particle)
Thermal correction to Energy= 0.433162
Thermal correction to Enthalpy= 0.434106
Thermal correction to Gibbs Free Energy= 0.339521
Sum of electronic and zero-point Energies= -1639.594315
Sum of electronic and thermal Energies= -1639.564314
Sum of electronic and thermal Enthalpies= -1639.563369
Sum of electronic and thermal Free Energies= -1639.657955

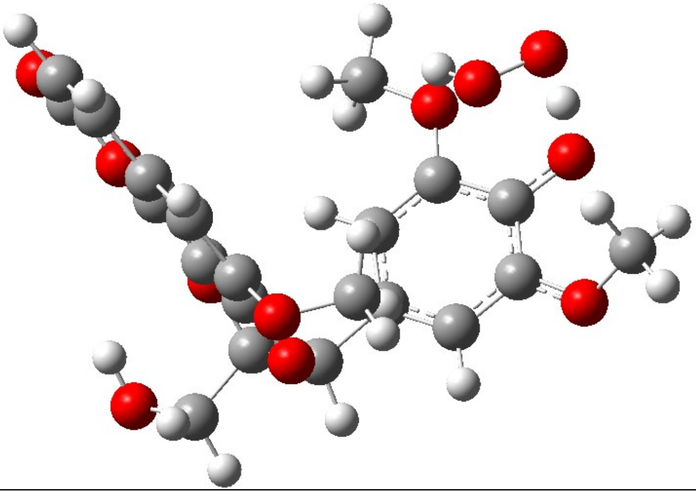

C 3.39676600 0.43205000 -0.82325000	
C -3.78262000 1.95814700 1.90802000	
C -4.42824000 2.57945500 0.90489000	
C -4.24909300 2.16458300 -0.48097800	
C 0.72752100 -1.20568900 3.32013600	
C 0.85123500 2.55031600 -2.35904200	
C 5.50246800 -0.40597300 1.02973000	
H 0.46026100 -3.33861700 -0.83434000	
H -0.72971100 -2.01254800 -2.66980400	
H -1.57195100 -4.20962000 -1.87413400	
H -2.46318900 -3.40520900 -0.56736400	
H 2.56879200 -2.72068000 0.15804800	
H 0.10047900 0.24355900 -1.76106100	
H -2.28089900 0.45760900 3.67670200	
H -3.92114800 2.27397700 2.93687500	
H -3.56533800 -2.26033400 -2.30128500	
H -5.10508100 3.40739000 1.06646900	
H 1.17848100 -1.85361600 4.07009200	
H 0.83768900 -0.15970200 3.62200800	
H 1.21616200 -1.36492300 2.35545400	
H 0.59544300 1.95352000 -3.23715200	
H 1.03428000 3.58038700 -2.65517900	
H 0.03433400 2.51398700 -1.63230000	
H 6.02654500 -1.02451500 1.75705500	
H 4.89534200 0.33731100 1.55088900	
H 6.21707400 0.09044900 0.37613400	
O 2.81379200 2.48834500 1.01721300	
H 2.13514000 2.73528000 0.36430000	
O 3.96629700 3.00246800 0.51744300	
H 4.29653400 2.14977800 -0.39343700	
**Name**	**CMC-C18-TS-P**
Cartesian Coordinates	Energy
O -0.19038000 -0.13934100 -1.78158100	Zero-point correction= 0.402034 (Hartree/Particle)
O -1.01535900 1.60430900 0.28216200	Thermal correction to Energy= 0.432729
O -1.51209400 3.87454100 -1.31857200	Thermal correction to Enthalpy= 0.433673
O -3.25409200 1.08259200 1.63739800	Thermal correction to Gibbs Free Energy= 0.337433
O -1.61828100 -2.23024200 -2.43065100	Sum of electronic and zero-point Energies= -1639.585661
O 4.63389800 1.14653300 1.70563300	Sum of electronic and thermal Energies= -1639.554966
O 2.84503200 -3.09269700 0.75537400	Sum of electronic and thermal Enthalpies= -1639.554022
O 4.69492600 -1.46613100 1.98848700	Sum of electronic and thermal Free Energies= -1639.650262
O -4.61368100 1.72672700 3.25095500	
C 0.68200500 0.80644000 -1.22411200	
C -0.07675900 2.02271000 -0.72522800	
C 1.70595800 0.20716700 -0.34258300	
C -1.73133000 0.50093300 -0.05827500	
C -1.33476600 -0.35602700 -1.07425400	
C -0.82058300 2.75704000 -1.83347000	
C -2.90571600 0.21613300 0.64937800	
C -2.10944100 -1.48835900 -1.41774400	
C 1.77849800 -1.17583500 -0.17475600	
C 2.66360300 1.03877000 0.27064400	
C -3.66796900 -0.89986800 0.33237500	
C -3.26651900 -1.75352000 -0.71280000	
C 3.65315500 0.47360600 1.05426400	

C	2.77175700	-1.73820400	0.61974300
C	3.71811700	-0.92132200	1.23530200
C	-4.86178000	-1.11197800	1.11184200
C	-5.20316500	-0.25698000	2.09278000
C	-4.38462900	0.90974700	2.39846100
C	-2.36321500	-3.37785400	-2.81174400
C	4.63638900	2.56661200	1.61529700
C	2.46051000	-3.56672600	2.04849400
H	0.58853700	2.71744500	-0.21531700
H	-0.09762900	3.13345900	-2.55993000
H	-1.50291400	2.07543300	-2.35600000
H	1.07071800	-1.83580500	-0.66091100
H	2.62986200	2.11106300	0.12553000
H	-3.87832300	-2.61522000	-0.94400800
H	-5.48002100	-1.97579300	0.88980900
H	-2.10716300	3.56500700	-0.62605700
H	-6.09089900	-0.38350900	2.69744200
H	-1.81370300	-3.83058400	-3.63396100
H	-3.36514400	-3.09906400	-3.15074100
H	-2.43531700	-4.09015900	-1.98489100
H	5.24573300	-0.74970700	2.33410400
H	4.78193300	2.88806300	0.58055500
H	5.47218800	2.90564200	2.22293300
H	3.70363200	2.97863800	2.00884500
H	2.56676800	-4.65000900	2.02397700
H	1.41782000	-3.30428600	2.25100200
H	3.10595200	-3.15407000	2.82642000
O	3.14276700	0.48726800	-2.99244400
H	3.74472200	0.71693900	-2.26559700
O	2.26089200	1.53750900	-3.05425800
H	1.38950600	1.23307700	-2.22492300

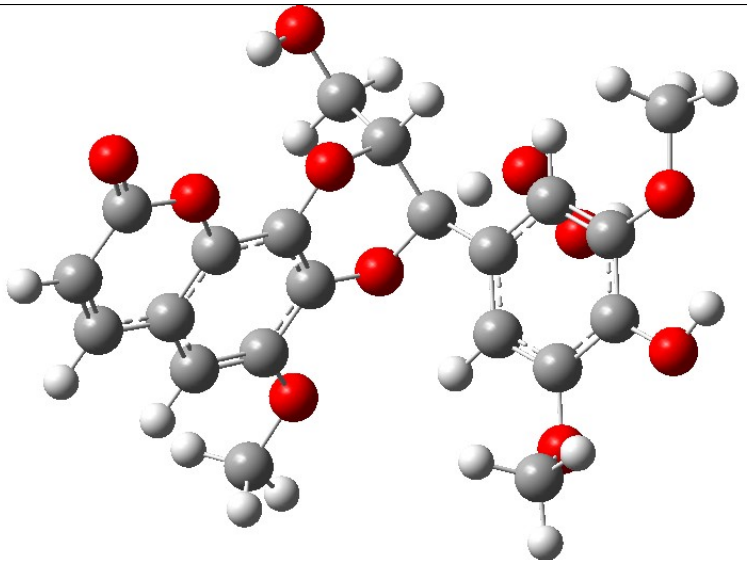

**Name**	**CMA-O14-TS-H**
Cartesian Coordinates	Energy

O	-0.26503300	0.96316700	-0.16940800
O	-1.77963500	-1.42839800	-0.24194600
O	0.25241800	-3.14115200	0.87828600
O	-4.43942000	-1.30016400	-0.08305300
O	-1.50200500	3.35978400	-0.02290200
O	5.10903600	-1.17489100	0.90909100
O	5.72652400	-0.16686700	-1.46169800
O	-6.32048100	-2.44414500	-0.06848700
C	0.33424000	-0.31530800	0.10215000
C	-0.41493100	-1.39254100	-0.68629300
C	1.78066000	-0.23660300	-0.29673600
C	-2.36080800	-0.20341900	-0.13057600
C	-1.61966600	0.97239500	-0.09817400
C	0.13908100	-2.78858000	-0.49151000
C	-3.75151100	-0.12032900	-0.04875000
C	-2.26905800	2.21950400	-0.00840700
C	-4.40152700	1.10659500	0.04939400
C	2.75001800	-0.73287900	0.56483300
C	2.12779600	0.28931700	-1.55385700
C	-3.64046900	2.28647800	0.06632600
C	4.08919500	-0.72046200	0.17348800

Zero-point correction=	0.368937 (Hartree/Particle)
Thermal correction to Energy=	0.396646
Thermal correction to Enthalpy=	0.397591
Thermal correction to Gibbs Free Energy=	0.308142
Sum of electronic and zero-point Energies=	-1525.127556
Sum of electronic and thermal Energies=	-1525.099846
Sum of electronic and thermal Enthalpies=	-1525.098902
Sum of electronic and thermal Free Energies=	-1525.188351

C	3.44976300	0.33361800	-1.93842000
C	-5.83848400	1.08383600	0.11574200
C	4.45192900	-0.17690100	-1.09575400
C	-6.51206200	-0.08240100	0.08040000
C	-5.80911400	-1.34476000	-0.02592200
C	-0.96291000	3.68768500	1.26697300
C	4.82855500	-1.62919300	2.23601600
H	0.23734700	-0.51967800	1.17180800
H	-0.39862700	-1.13355800	-1.75126700
H	-0.50541500	-3.49311000	-1.02467900
H	1.13944700	-2.84062700	-0.92214400
H	2.45815100	-1.12708300	1.52989600
H	1.35340600	0.66948400	-2.20953900
H	-4.12991000	3.25068900	0.13817400
H	3.75046900	0.74529700	-2.89449800
H	-6.36788500	2.02739800	0.19283100
H	-0.63519400	-3.18368600	1.25396500
H	-7.59079600	-0.13914600	0.12625200
H	-0.35130200	4.57724100	1.13005100
H	-0.34701700	2.86865100	1.64471900
H	-1.77577400	3.89750600	1.96621200
H	4.38080300	-0.82447300	2.82247000
H	4.16476000	-2.49534300	2.21025400
H	5.78924300	-1.90982700	2.65924800
H	6.26768000	0.48503400	-0.78255700
H	4.98053900	2.48033800	0.00809700
O	6.55571600	1.46790900	0.06660500
O	5.33236700	1.77083100	0.57415900

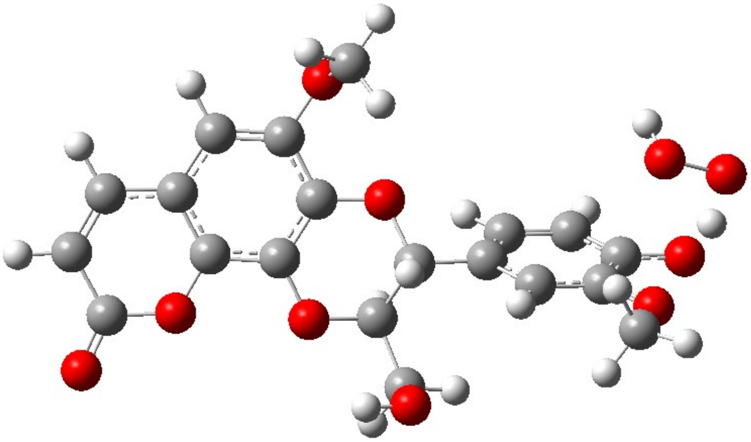

**Name**	**CMB-O14-TS-H**
Cartesian Coordinates	Energy

Zero-point correction= 0.369103 (Hartree/Particle)
Thermal correction to Energy= 0.396581
Thermal correction to Enthalpy= 0.397525
Thermal correction to Gibbs Free Energy= 0.309008
Sum of electronic and zero-point Energies= -1525.127689
Sum of electronic and thermal Energies= -1525.100211
Sum of electronic and thermal Enthalpies= -1525.099267
Sum of electronic and thermal Free Energies= -1525.187784

O	0.46283200	0.23358400	-0.30011100
O	1.76532200	-2.25349700	0.05622700
O	1.90922300	2.47226800	-0.36130800
O	-0.13954800	-3.86862900	-1.34805700
O	4.45957900	-2.37600100	0.21943700
O	-4.15731000	0.55149300	1.78516400
O	-5.73441400	-0.43402100	-0.10076200
O	1.86350500	4.66467600	-0.55394900
C	-0.17426200	-1.00371200	-0.65649100
C	0.35185700	-2.10831300	0.26454300
C	-1.65868500	-0.81666900	-0.51895100
C	1.81419100	0.13196900	-0.16884100
C	2.45693800	-1.08920100	-0.00052400
C	-0.25829100	-3.47038600	0.00833800
C	2.58414200	1.29518000	-0.20425900
C	3.86115400	-1.14447800	0.09886000
C	3.97102600	1.25184700	-0.09317100
C	-2.16783600	-0.17948300	0.60721200
C	-2.51311500	-1.32780000	-1.50949000
C	4.60813300	0.00921300	0.05578100
C	-3.54730000	-0.04818900	0.75364800
C	-3.87825600	-1.18919100	-1.38230300
C	4.67261100	2.50691900	-0.14796400
C	-4.42391700	-0.55700000	-0.25281300

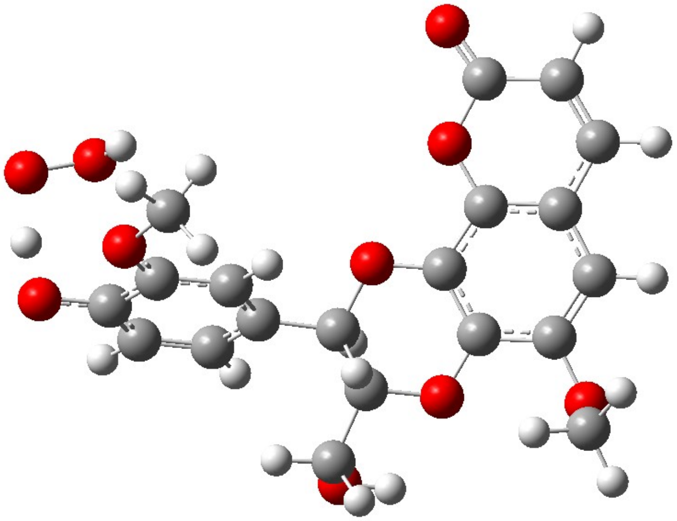

C	4.00179900	3.66528800	-0.29937500
C	2.55757700	3.67966300	-0.41387900
C	4.49313800	-2.84934500	1.57460700
C	-3.33744800	1.21274500	2.75368700
H	0.08778900	-1.24332300	-1.69062300
H	0.17855900	-1.81384800	1.30616700
H	0.22395200	-4.19413400	0.67157000
H	-1.32213800	-3.43977800	0.24551400
H	-1.49265200	0.20967900	1.35825300
H	-2.08959200	-1.81836000	-2.37807300
H	5.68732700	-0.04449800	0.13954100
H	-4.56021800	-1.55995500	-2.13806200
H	5.75407100	2.49920600	-0.06458600
H	0.79822400	-3.97289600	-1.54986700
H	4.49369300	4.62700300	-0.34456100
H	4.94589000	-3.83846900	1.54663400
H	3.48190000	-2.91579300	1.98201500
H	5.09957300	-2.17836300	2.18751000
H	-4.02701600	1.67225100	3.45661900
H	-2.70204000	0.49201600	3.27133100
H	-2.72715400	1.97855500	2.27002800
H	-5.98593800	0.62519800	-0.09576700
H	-4.22208300	2.42799800	0.08278500
O	-5.98364400	1.93051700	-0.31093100
O	-4.70840600	2.16375800	-0.71922500

**Name**	**CMC-O14-TS-H**
Cartesian Coordinates	Energy

Zero-point correction= 0.402064 (Hartree/Particle)
Thermal correction to Energy= 0.432288
Thermal correction to Enthalpy= 0.433233
Thermal correction to Gibbs Free Energy= 0.338317
Sum of electronic and zero-point Energies= -1639.602701
Sum of electronic and thermal Energies= -1639.572476
Sum of electronic and thermal Enthalpies= -1639.571532
Sum of electronic and thermal Free Energies= -1639.666448

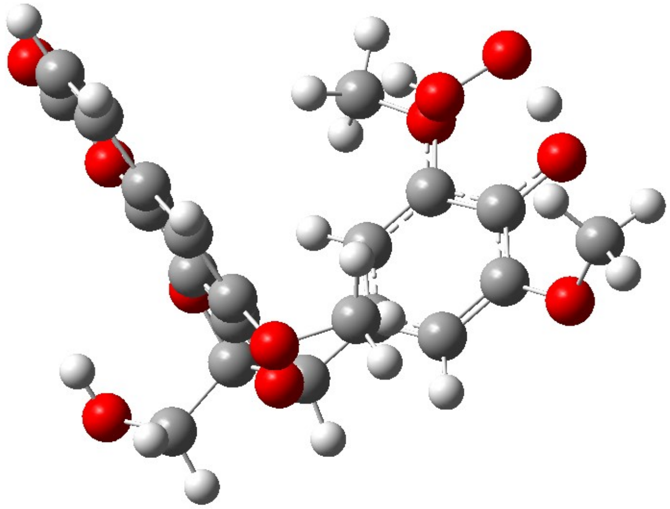

O	-0.37282700	-2.44498500	0.51203100
O	-1.79748200	-0.87370700	-1.36926400
O	-3.24055900	-3.00920400	-2.56992100
O	-3.19578400	1.22277600	-0.53764700
O	-0.45167300	-1.81712300	3.14616100
O	2.15320100	1.93378900	-2.21791600
O	4.45838800	-1.07298300	0.63977300
O	4.32386900	1.31171300	-0.85599200
O	-4.48540200	2.90250800	-1.13852800
C	0.06077200	-2.37094400	-0.86314200
C	-1.14747700	-2.10105900	-1.74921000
C	1.19514400	-1.36668000	-0.95027500
C	-1.80713300	-0.60547000	-0.03683300
C	-1.10459400	-1.36220400	0.89537000
C	-2.15252400	-3.24087300	-1.69119400
C	-2.53575100	0.49684200	0.41339400
C	-1.14986800	-1.02993300	2.26185800
C	2.33628500	-1.65988100	-0.19607900
C	1.08945800	-0.17165000	-1.65874700
C	-2.58812300	0.83231000	1.76308100
C	-1.88570900	0.04982900	2.69304900
C	2.14131100	0.73747900	-1.60764400
C	3.37299500	-0.75114200	-0.11182700
C	3.31159000	0.45985600	-0.84435800
C	-3.37343000	1.98418500	2.12204700
C	-4.01874700	2.69857000	1.18021800
C	-3.94394500	2.32636100	-0.21857000

C	0.90331500	-1.37490200	3.32411100	
C	0.95876500	2.35594600	-2.88102200	
C	4.95625500	-0.07202300	1.54765900	
H	0.44008500	-3.36840500	-1.08661100	
H	-0.82744400	-1.94801800	-2.78084500	
H	-1.66033900	-4.15724200	-2.01989800	
H	-2.50836500	-3.38237300	-0.66524100	
H	2.40264600	-2.58560900	0.36425500	
H	0.20168100	0.05714300	-2.23052100	
H	-1.91300900	0.29882500	3.74751500	
H	-3.43207300	2.26426500	3.16829900	
H	-3.73018700	-2.24402600	-2.24576000	
H	-4.61594500	3.56991500	1.41084900	
H	1.37769400	-2.08773400	3.99586600	
H	0.91402400	-0.37697400	3.76937200	
H	1.42864000	-1.35934000	2.36543900	
H	0.73033500	1.69281400	-3.71729600	
H	1.16550000	3.35773100	-3.24811800	
H	0.12317500	2.37938900	-2.17726200	
H	5.34641200	-0.61617200	2.40624000	
H	4.14633500	0.58629400	1.86721000	
H	5.75098400	0.50837300	1.08269200	
O	2.29569200	2.31621800	0.86324700	
H	1.61541900	2.59951900	0.22411200	
O	3.41158500	3.01523200	0.54530900	
H	4.03618200	2.19481800	-0.29707800	

References

1. M. G. Evans and M. Polanyi, *Trans. Faraday Soc.*, 1935, **31**, 875-894.
2. H. Eyring, *J. Chem. Phys.*, 1935, **3**, 107-115.
3. D. G. Truhlar, W. L. Hase and J. T. Hynes, *J. Phys. Chem.*, 1983, **87**, 2664-2682.
4. T. Furuncuoglu, I. Ugur, I. Degirmenci and V. Aviyente, *Macromolecules*, 2010, **43**, 1823-1835.
5. E. Vélez, J. Quijano, R. Notario, E. Pabón, J. Murillo, J. Leal, E. Zapata and G. Alarcón, *J. Phys. Org. Chem.*, 2009, **22**, 971-977.
6. E. Pollak and P. Pechukas, *J. Am. Chem. Soc.*, 1978, **100**, 2984-2991.
7. A. Fernández-Ramos, B. A. Ellingson, R. Meana-Pañeda, J. M. Marques and D. G. Truhlar, *Theor. Chem. Acc.*, 2007, **118**, 813-826.
8. C. Eckart, *Phy. Rev.*, 1930, **35**, 1303.
9. R. A. Marcus, *Annu. Rev. Phys. Chem.*, 1964, **15**, 155-196.
10. R. A. Marcus, *Rev. Mod. Phys.*, 1993, **65**, 599.
11. Y. Lu, A. Wang, P. Shi and H. Zhang, *PloS one*, 2017, **12**, e0169773.
12. Y. Lu, A. Wang, P. Shi, H. Zhang and Z. Li, *PloS one*, 2015, **10**, e0133259.
13. S. F. Nelsen, S. C. Blackstock and Y. Kim, *J. Am. Chem. Soc.*, 1987, **109**, 677-682.
14. S. F. Nelsen, M. N. Weaver, Y. Luo, J. R. Pladziewicz, L. K. Ausman, T. L. Jentzsch and J. J. O'Konek, *J. Phys. Chem. A*, 2006, **110**, 11665-11676.
15. A. Galano and J. R. Alvarez-Idaboy, *J. Comput. Chem.*, 2013, **34**, 2430-2445.
16. F. C. Collins and G. E. Kimball, *J. Colloid Sci.* , 1949, **4**, 425-437.
17. M. Von Smoluchowski, *Z. Phys. Chem*, 1917, **92**, 129-168.
18. D. G. Truhlar, *J. Chem. Educ.*, 1985, **62**, 104.
19. A. Einstein, *Ann. Phys.*, 1905, **17**, 549-560.
20. G. G. Stokes, *Mathematical and Physical Papers*, University Press, Cambridge, 1905.
21. A. Galano and J. Raúl Alvarez-Idaboy, *Int. J. Quantum Chem.*, 2019, **119**, e25665.
22. Q. V. Vo, T. V. Gon, M. V. Bay and A. Mechler, *J. Phys. Chem. B*, 2019, **123**, 10672-10679.
23. Q. V. Vo and A. Mechler, *J. Chem. Inf. Model.*, 2020, **60**, 316-321.
24. Y. Okuno, *Chem.: Eur. J.*, 1997, **3**, 212-218.
25. S. Benson, *The foundations of chemical kinetics:* , Malabar, Florida, 1982.
26. C. Iuga, J. R. Alvarez-Idaboy and A. Vivier-Bunge, *J. Phys. Chem. B*, 2011, **115**, 12234-12246.
27. J. R. Alvarez-Idaboy, L. Reyes and N. Mora-Diez, *Org. Biomol. Chem.*, 2007, **5**, 3682-3689.
28. T. H. Le, T. T. Tran and L. K. Huynh, *Chemom. Intell. Lab. Syst.*, 2018, **172**, 10-16.
29. E. Dzib, J. L. Cabellos, F. Ortíz-Chi, S. Pan, A. Galano and G. Merino, *Int. J. Quantum Chem.*, 2019, **119**, e25686.

30. E. Dzib, J. L. Cabellos, F. Ortiz-Chi, S. Pan, A. Galano and G. Merino, *Eyringpy 1.0.2*, 2018, Cinvestav, Mérida, Yucatán.
